# Talk-based approaches to support people who are distressed by their experience of hearing voices: A scoping review

**DOI:** 10.3389/fpsyt.2022.983999

**Published:** 2022-10-10

**Authors:** Christian Burr, Joachim K. Schnackenberg, Frank Weidner

**Affiliations:** ^1^Department of Health Professions, Bern University of Applied Sciences, Bern, Switzerland; ^2^University Hospital of Psychiatry and Psychotherapy, University Hospital for Mental Health, Bern, Switzerland; ^3^Faculty of Nursing Science, Vinzenz Pallotti University, Vallendar, Germany; ^4^efc-Institut, Kropp, Germany

**Keywords:** hearing voices, auditory hallucination, mental health, intervention, approach, transdiagnostic, nursing, professionalization

## Abstract

**Background:**

The positive effects of both antipsychotic medication and cognitive behavioral therapy in psychosis (CBTp) for people who are distressed by their experience of hearing voices remain limited. As a result, there has been a recent surge in talk-based individual approaches. Many of these continue not to be very well known nor implemented in practice. Some of the approaches may focus more on understanding and dealing constructively with voices, an element that has been identified as potentially helpful by voice hearers. Existing barriers to a wider implementation include both the widespread pathologization of hearing voices and a lack of mental health professionals who have been trained and trusted to carry out these new interventions.

**Methods:**

This scoping review aimed to identify and describe a current synthesis of talk-based individual approaches for people who hear voices, including studies independently of method of study or approach, diagnosis of voice hearers nor of the professional background of interventionists.

**Results:**

Nine different talk-based approaches were identified. These included: (1) Cognitive Behavioral Therapy for Psychosis (CBTp); (2) AVATAR therapy; (3) Making Sense of Voices (MsV) aka Experience Focused Counselling (EFC); (4) Relating Therapy; (5) Acceptance and Commitment Therapy; (6) Smartphone-based Coping-focused Intervention; (7) Prolonged and Virtual Reality Exposure Therapy; (8) Eye Movement Desensitization and Reprocessing, and (9) Individual Mindfulness-based Program for Voice Hearing. The different approaches differed greatly in relation to the number of sessions, length of time offered and the scientific evidence on efficacy. Psychologists represented the main professional group of interventionists. CBTp and the MsV/EFC approach also included health professionals, like nurses, as implementers. Most of the approaches showed positive outcomes in relation to voice related distress levels. None identified overall or voice specific deteriorations.

**Conclusion:**

There appears to be a strong case for the implementation of a broader heterogeneity of approaches in practice. This would also be in line with recommendations for recovery focused services and requirements of voice hearers. A greater emphasis on whole systems implementation and thus the involvement of frontline staff, like nurses, in the delivery of these approaches would likely reduce the research-practice implementation gap.

## Introduction

Cognitive Behavioral Therapy in Psychosis (CBTp) has long been considered the main or only talk-based approach for people who are distressed by their experience of hearing voices in national guidelines (1), even though other approaches do exist (2, 3). This may well be a direct result of CBTp researchers' widespread use of more widely accepted forms of evidence gathering, primarily the high number of randomized controlled trials. This contrasts to the still developing evidence base of other new approaches (3) and the relative lack of positive outcomes and use of those forms of evidence in relation to older approaches, like psychodynamic therapy (2). Despite the large number of studies, the positive effects of CBTp (4, 5) and antipsychotic medication use (5–7) seem to be small to medium only. As a result there has been a recent surge in talk-based individual approaches using communication and psychological theories to support people distressed by their experience of hearing voices (3). The authors use the term “approaches” rather than “therapies” as a generic term to include not only the methods and intervention tools used but also incorporate the attitude and philosophy required. In addition, it was important to include all approaches in one term, including those that did not consider themselves specifically a therapy, such as Making Sense of Voices (MsV) /Experience Focused Counseling (EFC) or Eye Movement Desensitization and Reprocessing (EMDR). In the same vein, we used the term interventionists—rather than therapists—to include all approaches. These new approaches include acceptance- and mindfulness-based methods as well as new ways of communicating with and about the voices, learning from the relationship with the voices and the environment, and working with the personal history (8). Additionally, trauma-focused approaches are described and recommended (9). Talk-based approaches like trauma-focused therapies and CBT that do not explicitly focus on voice-hearing have also shown positive results in relation to voice-hearing (10, 11). Within most of these new approaches, there is an emphasis on self-management, understanding or on learning to deal more constructively with the voices. Expanding on the approaches available is also in line with the wishes and demands made on mental health services by people who hear voices and are distressed by them (12, 13) as well as by family members (14).

The experience of voice-hearers shows that talk-based approaches with a focus on understanding and dealing constructively with voices are not yet commonplace in psychiatric practice. For example, classic reactions by professionals to voices can include attempts to suppress and pathologize the experience and thus contribute to people who hear voices feeling discouraged from talking about them (13, 15). This paradigm of discouraging open talk about the voice hearing experience has been dominant in mainstream mental health services for many decades (13, 16, 17). As a result, only some people with a diagnosis of psychosis and the experience of hearing voices currently have access to recently developed talking approaches (18–20).

There may be a variety of reasons for the apparent discrepancy between the nature and number of various talk-based approaches and what seems to be common psychiatric practice. One of the barriers to a wider practice implementation may be, for example, the relative lack of knowledge and training of mental health professionals (8, 21). Regulations regarding the requirements for offering, carrying out and invoicing such approaches vary worldwide and in Europe, too (22). Some countries stipulate that only certain psychotherapists are traditionally entrusted with the implementation of psychotherapeutic approaches (e.g., in Germany, Italy, and Switzerland). In others, a shorter specific training is sufficient, which can also be completed by other mental health professionals, such as nurses or social workers (e.g., in The Netherlands, Finland, and Austria). In the UK there appears to be a higher acceptance of therapeutic work being undertaken by various professions working within mainstream psychiatric services even if they are not formally trained clinical psychologists. One initiative, for example, the Improving Access to Psychological Therapies (IAPT) program (23), which was launched to meet the increasing demand for psychotherapeutic therapies being offered for various forms of depression and anxiety, specifically promotes interventions being offered by a variety of health professionals trained in specific therapy modalities. It thus opens up greater access to therapists who do not have to have undergone the highly rigorous, competitive and demanding training to become a doctorate level clinical psychologist first. Availability of support in everyday life situations also seem to be important (8). With their focus on collaborative support in everyday life situations and relation building (24), nursing professions and similarly working professionals appear predestined for the implementation and offering of psychosocial therapeutic approaches in everyday life.

Current reviews on talk-based approaches for people who are distressed by their voice hearing experience focus on specific approaches and diagnoses. They also do not contain the latest developments in talk-based approaches. Existing reviews focused either on specific therapy approaches, such as CBTp (25) or Acceptance and Commitment Therapy (ACT) (26), on specific diagnoses (generally psychosis or schizophrenia) (27, 28) or presentations, such as trauma (10). In a very comprehensive report of a research consortium on psychological approaches to voice hearing (8) new talk-based approaches like Relating Therapy and the Audio-Visual Assisted Therapy Aid for Refractory auditory hallucinations (AVATAR therapy) or the approach developed by the Hearing Voices Movement (HVM) were only briefly described, as classic intervention studies relating to these approaches had not been completed at the time.

The aim of the present scoping review was therefore to identify and describe a current overview of all studies identified in the literature on talk-based individual approaches for people who hear voices and are distressed by that experience. The focus of this review on individual approaches honors the fact that 1-to-1 situations, within which these approaches might helpfully be implemented, are already a possibility and in part common practice for frontline staff, such as nurses, within existing structures and thus promises to improve access to appropriate psychosocial approaches. As many of the approaches are fairly new in their evidence base development but are claiming to be effective for voices the main outcome focus needed to be on the approaches' effect on voices or on related experiences or phenomena, such as psychosis. An additional focus of this review was on the description of the content of the intervention part of the approach, its effectiveness, and the experience of it, as well as on the identification of the professional background of the persons who implemented the approaches.

## Methods

### Design

We chose a scoping review design to provide a broad overview of talk-based approaches to voice hearing, regardless of the methodology or the quality of the studies. This allowed for a content, trans-diagnostic (as similar voice hearing experiences are reported trans diagnostically) (29), and trans-methodic focus, as well as for the inclusion of both quantitative and qualitative research designs (30, 31). This scoping review followed the steps suggested by the Joanna Briggs Institute (32): (a) define and align the objectives and questions (as outlined in the introduction); (b) define inclusion criteria; (c) describe the planned approach to searching, selecting, extracting and presenting evidence; (d) search for evidence; (e) select the evidence; (f) extract the evidence; (g) present the evidence graphically; and (h) summarize the evidence in relation to objectives and questions.

### Search strategy

The search was conducted on January 24, 2021 in the following databases: MEDLINE (Pubmed), Embase, Cochrane Library, CINAHL, PsycINFO, and Psyndex and included all search results in English and German for the years 1990–2021 to include the very first years of CBTp research Garety et al. (33) and the specific research on hearing voices by Romme and Escher (34), as the co-founders of the Hearing Voices Movement. The search combined the following terms: (“hearing voices” OR “acoustic hallucination” OR “auditory hallucination” OR “psychosis^*^” OR “psychotic^*^”) AND (“intervention” OR “therapy” OR “counseling”) NOT (“Transcranial Magnetic Stimulation” OR “neuroleptic^*^” OR “antipsychotic^*^” OR “music^*^” OR “art therapy”).

To complete the search, a Google, and Google Scholar search, a hand search by the two authors (CB, JS) in their own filing system, and a review of the reference lists of the included articles were also performed. Documentation of the detailed search strategy by database can be requested from the first author.

### Inclusion and exclusion criteria

Studies had to fulfill the following criteria to be included:

- the approach had to be a talk-based individual therapy or have a counseling focus or a talk-based instruction for an individual intervention
- qualitative or quantitative intervention study types in English or German
- the intervention referred to voice hearing or auditory hallucinations as a target
- voice hearing, auditory hallucinations or positive symptoms are described as an outcome criterion of the intervention
- study participants ≥18 and ≤65 years

The following exclusion criteria applied:

- group intervention
- mixed group and individual or multimodal intervention design
- studies with <4 participants (e.g., single case studies)
- study protocols
- non talk-based intervention

Two additional exclusion criteria were defined for the review of the full texts:

- single study is part of an included systematic review and/or meta-analysis or synthesis
- existence of a more recent version of an included systematic review and/or meta-analysis or synthesis on a similar or same research question.

### Study selection

Two authors (CB, JS) independently performed a title and abstract screening, which was then followed by a full text review. Studies without a mutual match were decided on by consensus by both reviewers using the in- and exclusion criteria. Finally, all review articles (systematic reviews, meta-analyses, or -syntheses) with similar questions were identified and only the most recent ones were included. Individual studies that were included in one or more review articles were identified and excluded. Data management was performed with endnote 20 (35).

### Data extraction

Data extraction was performed by one research team member (CB) and checked for accuracy by a second person (JS). For an initial analysis of the studies, the following information was extracted: study identifier (author and year); the study's country location; number of studies and/or study participants included; symptom or diagnostic focus of the approach; name of the approach; number of sessions of the approach conducted over what period in months; control condition for controlled design; method or design; measurement tools used and results (statistical significance and effect sizes related to voice hearing, and if not reported, to positive symptoms between baseline and post-intervention or follow-up). The focus on voice specific or voice associated (like positive symptoms) distress levels and experiences was chosen as this review specifically aimed to provide an overview of the various talking approaches on their stated target. Most of the approaches are still developing their evidence base and would thus benefit from comparing their effects (both quantitative and qualitative) with their stated aims (voice related distress reduction). Effect sizes (ES) ranges for Cohen's “*d*” or Hedge's “g” were defined as “low” = 0.2, “medium” = 0.5, and “large” = 0.8 (36). Studies with an explicit focus on hearing voices were further examined for: content; professional background, qualification, training, and supervision of the professionals carrying out the approaches.

## Results

### Study selection

**Figure 1:**
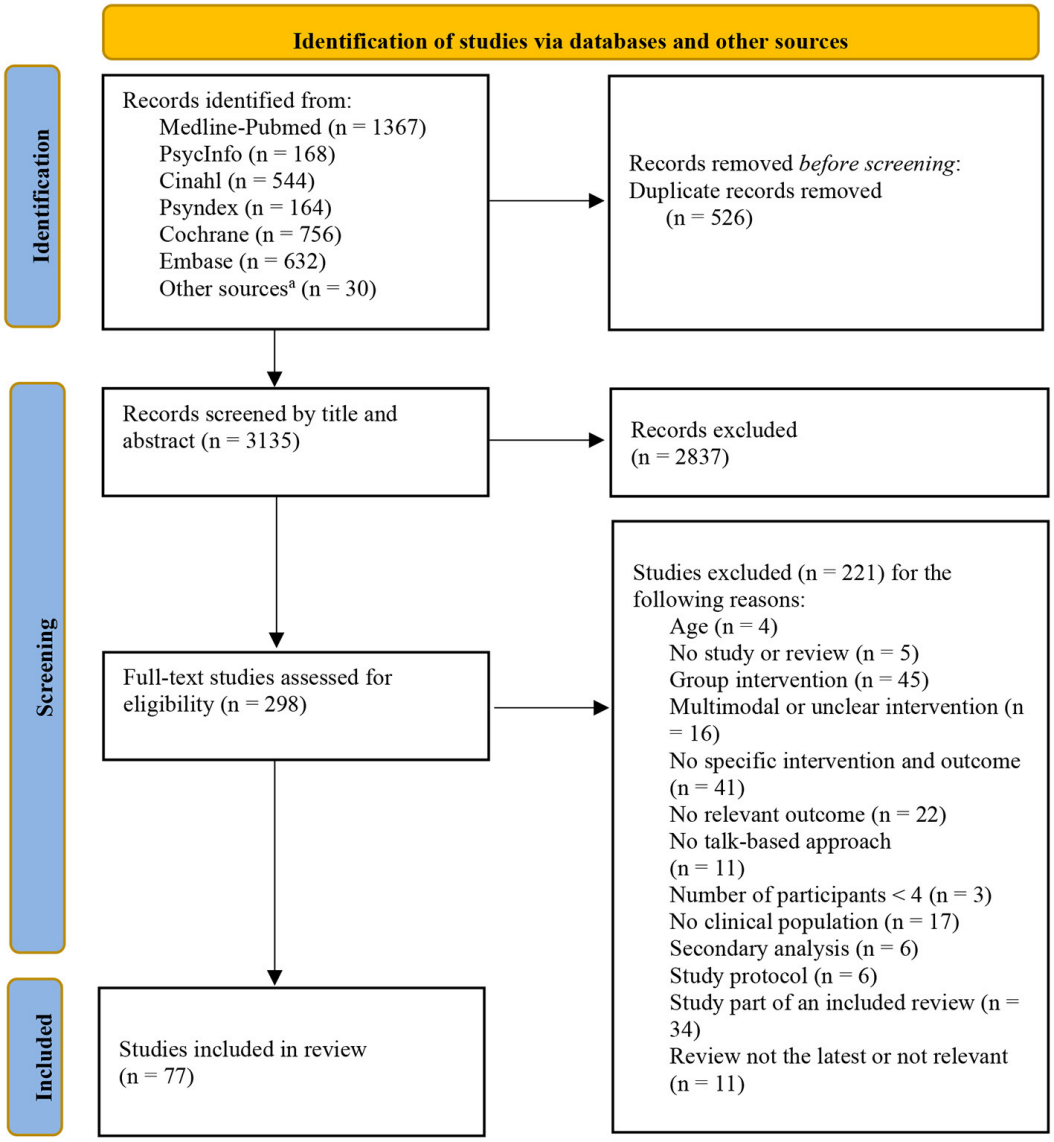
Adapted PRISMA 2020 flow diagram for Systematic Reviews of the study selection process. ^a^Reference lists from included articles, Google search, and personal storage.

The systematic Boolean/phrase title search strategy identified 3,622 title references. A further 30 titles were found through other sources such as Google, Google-Scholar, reference lists of included articles or personal files of the authors. After removal of duplicates, a title and abstract screening was performed for 3,135 hits leaving 298 full texts to review to check eligibility, leading to 77 articles meeting the inclusion criteria (Figure 1). A tabular overview of the analysis of all studies is available as Supplementary Table S1.

### Publication date and study location

16.9% (*n* = 13) of the included studies were published before 2010, about one third in the following 5 years (*n* = 25; 32.5%), a further 36.4% (*n* = 28) between 2015 and 2019 and 14.3% (*n* = 11) from 2020 onwards. Most studies were from the UK (*n* = 35; 45.5%), followed by Australia (*n* = 10; 13.0%), Germany (*n* = 9; 11.7%), the USA (*n* = 6; 7.8%) and the Netherlands (*n* = 5; 6.5%). The remaining 12 studies (15.6%) were distributed among seven other countries (Canada, Norway, Denmark, Hongkong, Turkey, Poland, and Austria).

### Study designs

Most of the included studies had a quantitative design (*n* = 69; 89.6%). Of these, 56 (81.2%) were single studies and 34 (49.3%) had a randomized-controlled design. Systematic reviews and or meta-analyses made up 13 (18.8%) studies. Seven studies had a qualitative design (9.1%) including one qualitative meta-synthesis. One study had a mixed-methods design.

### Focus of the intervention and measurements

Nine studies (11.7%) had voice hearing as the primary focus of the approach independently of diagnosis. A further 25 (32.5%) reported voice hearing within the context of diagnosis or presentation (e.g., psychosis or trauma) or in combination with other symptoms or phenomena (e.g., delusions *aka* non-shared realities) (see Table 1). In all other studies, the approach was not directly related to voice hearing/auditory hallucinations but an outcome measure relating to voice hearing or auditory hallucinations, or positive symptoms of psychosis was, respectively, identified. Within the 69 quantitative design studies, voice hearing or auditory hallucinations were measured in 41 separate studies (59.4%) using 14 different instruments. The most frequently used instrument (*n* = 33; 80.5%) was the Auditory Hallucination Scale of the Psychotic Symptom Rating Scales (PSYRATS-AH) (37). The Beliefs About Voices Questionnaire—Revised (BAVQ-R) (38) and the Hamilton Program for Schizophrenia Voices Questionnaire (HPSVQ) (39) were used more than once and 11 others once each. Positive symptoms of psychosis or schizophrenia were measured in 41 studies using the Positive and Negative Syndrome Scale (PANSS) (40). In four studies, other instruments were used for this purpose. In two studies in which the approach focused on voice hearing, only general psychopathology was measured with two different instruments (Table 2).

**Table 1:** Overview of articles included in the synthesis.

**Study**			**Intervention^c^**	**Design^c^**
**Nr.^a^**	**References**	**Country^b^**		
1	Aali et al. (2020)	GBR, CAN	AVATAR therapy	Systematic review and meta-analysis
3	Bacon et al. (2013)	AUS	Acceptance and Commitment Therapy	Qualitative study
9	Bell et al. (2020)	AUS	Smartphone based, coping focused Intervention	RCT
11	Birchwood et al. (2018)	GBR	CBTp	Qualitative study
13	Brown et al. (2020)	AUS	Acceptance and commitment therapy	Systematic review and meta-analysis
14	Buck et al. (2019)	USA	Prolonged and virtual reality exposure	Controlled trial—WL design
15	Burns et al. (2014)	CAN	CBTp	Systematic review and meta-analysis
17	England (2007)	CAN	CBTp	RCT
21	Gould et al. (2001)	USA	CBTp	Systematic review and meta-analysis
24	Hayward et al. (2009)	GBR	Relating therapy	Case series
25	Hayward et al. (2017)	GBR	Relating therapy	RCT
28	Jolley et al. (2015)	GBR	CBTp	Observational study
29	Keen et al. (2017)	GBR	CBTp	Case series
31	Krakvik et al. (2013)	NOR	CBTp	RCT
33	Lewis et al. (2002)	GBR	CBTp	RCT
35	Lincoln et al. (2016)	DEU	CBTp	Observational study
36	Lincoln et al. (2019)	DEU	CBTp	Systematic review
37	Louise et al. (2019)	AUS	Mindfulness-based cognitive therapy	Observational study
39	Matthijsen et al. (2019)	NLD	Eye movement desensitization and reprocessing	Intervention study—within-subject design
40	Morrison et al. (2004)	GBR	CBTp	RCT
41	Morrison et al. (2012)	GBR	CBTp	Observational study
43	Morrison et al. (2014b)	GBR	CBTp	RCT
51	Schnackenberg et al. (2018)	DEU	Experienced focused counseling	Qualitative study
52	Schnackenberg et al. (2016)	DEU	Experienced focused counseling	RCT
53	Schnackenberg et al. (2018b)	DEU	Experienced focused counseling	Qualitative study
54	Schnackenberg et al. (2014)	DEU	Experienced focused counseling	Systematic review
56	Sivec et al. (2017)	USA	CBTp	Case comparison (retrospective)
58	Steel et al. (2019)	GBR	Making sense of voices (MsV)	Case Series with WL
59	Steel et al. (2020)	GBR	Making sense of voices (MsV)	Qualitative study
60	Stefaniak et al. (2019)	POL	AVATAR therapy	RCT
61	Tarrier et al. (2001)	GBR	CBTp	RCT
68	Turkington et al. (2014)	GBR	CBTp	Observational study
73	Varese et al. (2020)	GBR	CBTp	Case series
74	Velligan et al. (2015)	GBR	CBTp	RCT

a Corresponds with numbers on the table of the analysis of all included studies (Supplementary Table S1).

b ISO-Code.

c CBTp, Cognitive Behavioral Therapy for Psychosis; RCT, Randomized controlled trial; WL, Wait List.

**Table 2:** Outcome measurements used in the quantitative studies (*n* = 69).

**Variable**	***n***	**%**
**Studies used outcome measures for hearing voices (*****n*** **= 41)**		
PSYRATS-auditory hallucination subscale (PSYRATS-AH)^a^	33	80.5
Beliefs about voices questionnaire—revised (BAVQ-R)	3	7.3
Hamilton program for schizophrenia voices questionnaire (HPSVQ)	2	4.9
Others (once each)^a^: AHS, BASIS-24-Hall, DAIMON, PRS-Hall, PSE-Hall, SAPS-Hall, VAS-Coping, VAY, VCS, VPDS	11	26.9
**Studies used outcome measures for positive or psychotic symptoms (*****n*** **= 41)**		
Positive and negative syndrome scale for schizophrenia (PANSS) (without specification)	41	100.0
PANSS positive symptoms subscale	29	70.7
Others (once each)^a^: PSYRATS-General, SAPS, BPRS-E Psychosis, CAPE-Positive	4	9.8

a AHS, Auditory Hallucination Scale (AHS); BASIS-24-Hall, Behavior and Symptom Identification Scale, Hallucination Subscale; CAPE-Positive, Community Assessment of Psychic Experience Questionnaire—positive Symptoms; DAIMON-Scale to measure the dialogical characteristics of the relationship between the hearer and their voice(s); PSE-Hall, Present State Examination—Hallucination symptoms categories; PSYRATS-AH, Psychotic Symptoms Rating Scale; SAPS-Hall, Scale for the Assessment of Positive Symptoms—Hallucination Subscale; VAS-Coping, Visual Analog Scale—Coping with and understanding Voices; VAY, Voice and You–Questionnaire; VCS, Voice Compliance Scale; VPDS, Voice Power Differential Scale.

### Approaches with a focus on voice hearing

Among the included studies with voice hearing as the primary focus of the approach (*n* = 9), 4 (references to articles numbered as in Table 1 or Supplementary Table S2 are shown in square brackets: [52, 53, 58, 59]) applied the Making Sense of Voices approach aka Experience Focused Counseling (EFC) and 2 Relating Therapy [24, 25]. Another 3 investigated a smartphone-based, coping focused approach [9], one CBTp approach focused on commanding voices [11] and one on a form of EMDR (Eye Movement Desensitization and Reprocessing) for auditory hallucinations [39].

Of the studies which included hearing voices as one of several focuses of the approach (*n* = 25), 14 examined CBTp [15, 21, 28, 31, 33, 35, 36, 40, 41, 43, 56, 61, 68, 73]. One each applied cognitive adaptation training (CAT) [74], cognitive nursing interventions for hearing voices [17] and trauma-focused CBT [29]. Two studies each were using AVATAR therapy [1, 60], Acceptance and Commitment Therapy (ACT) [3, 13] and EFC with a focus on trauma [51, 54]. Two other individual studies investigated a Mindfulness-based Program for Voice Hearing (iMPV) [37] and Prolonged or Virtual Reality Exposure therapy (PE & VRE) [14].

To gain a more detailed understanding of the various approaches identified, the following section describes a synthesis of the respective contents, formal aspects, qualification, and training of the professionals, as well as results from the quantitative and qualitative studies (Table 3).

**Table 3:** Synthesis of studies of talk-based approaches with a primary focus on hearing voices.

**Name of the intervention**	**Studies** Number of studies Study numbers^a^ Number of participants	**Content of the intervention**	**Conducting the intervention** Number of sessions and duration of the intervention Professional background of the interventionists Training of the professionals	**If reported:** **Quantitative results related to voice hearing only** (if indicated, specific measurements are named; if no total scores available, subscales were used) Pre-, to post-test or pre-test to follow-up Significance level: *p* = < 0.05 Effect size: Hedges g or Cohens d **Qualitative results** Brief description
**CBTp** Cognitive behavioural therapy for psychosis	18 11, 15, 17^b^, 21, 28^b^, 29, 31^b^, 33, 35, 36, 40, 41, 43^b^, 56^b^, 61, 68, 73, 74 1932	**Relationship building and normalisation:** Development of an understanding about the symptoms and voices. **Psychoeducation:** Education about the nature, treatment, and relapse prevention of psychosis. **Case formulation:** conceptualising the voices based on cognitive models **Working through the voices and developing coping strategies:** Addressing the maintaining factors related to the voices, stresses or negative beliefs through cognitive behavioural techniques **Contents of specialised programmes** • Improving the link between therapy and everyday life • Techniques for people with dissociations • Exposure techniques for people with psychoses and PTSD	5–25 sessions over 1–9 months Psychologists with master's degree or doctorate, partly other professions like psychiatrists, occupational therapists, and nurses Specific training between 10 h, 5-day course and 120 h multimodal training Individual supervision 1 to 2-weekly	**Quantitative:** Significant improvement (28,29,31,33,40,41,43,61,73,74)^a^ Effect sizes: medium to large 0.56–1.08 (28,41,73)^a^ Effect sizes: small to large 0.3–1.4 (36)^a^ Effect sizes: small to medium 0.1–0.56 (31,56,68)^a^ **Qualitative:** More control and power over the voices, normalisation of the voice experience, fear of answering or contradicting the voices remains (11)^a^
**AVATAR therapy** Audio-visual assisted therapy aid for refractory auditory hallucinations	2 1, 60 218	**Creating an AVATAR**: a visual and audio representation (avatar) of the main voice is created on a computer screen and its voice is defined. **Dialogue with the AVATAR**: within 45-min sessions about 10-to-15-min are spent practising assertive communication between the voice hearing person and the avatar (who is voiced by the therapist). The therapist can sit in the same or another room as the voice-hearing person and supports and accompanies him/her in increasing assertive communication. **1st phase:** the avatar speaks the literal content of the voice in the way the voice hearing person is used to. The voice hearer is encouraged to practice a confident response to it. **2nd phase:** the avatar and his voice change in a constructive and supportive direction and the dialogue changes accordingly to become less confrontational.	6–7 weekly sessions over 2 months Therapists with experience in CBT Specific training not clearly defined, a manual should be used, weekly supervision with experienced therapist	**Quantitative:** Significant improvement (1,60)^a^ Effect sizes not reported
**Smartphone-based CFT** Smartphone-based coping-focused intervention for voice hearing	1 9 34	**The foundation of the intervention** is Coping Strategy Enhancement (CSE) which encourages the use of past useful and effective strategies only **First session:** Introduction and training on how to use the smartphone app and how to collect data on voices and strategies. **Monitoring phase:** collect data on voices and strategies over six days, evaluate data **Second session:** Identification and definition of alternative strategies to deal with the voices. **Monitoring and intervention phase:** Participants receive regular reminders to use strategies and continue to collect data regularly over 10 days. **A third session** is repeated, and the **monitoring and intervention phase** is further extended.	4 sessions over ~1 month. In between, practice the interventions with the support of the smartphone app. Doctorate in clinical psychology No information about specific training and supervision	**Quantitative:** *Voice hearing (PSYRATS-AH total)*: No significant improvement (9)^a^ Effect size medium: 0.55 (9)^a^ *Subjective assessment of coping (VAS*): Significant improvement (9)^a^ Effect size: high 1.45 (9)^a^ *Subjective appraisal of understanding the voices (VAS):* significant improvement (9)^a^ Effect size: medium 0.61 (9)^a^
**ACT** Acceptance and Commitment Therapy	2 3, 13 274	**Introduction:** ACT approach is introduced followed by the beginning of therapy: **Cognitive “defusion”:** therapist takes on the role of the voices or thoughts (mind), comments, evaluates, analyses things and events and gives recommendations for action, voice hearers observe what the mind does without communicating or following what it says or thinks, experience that controlling and suppressing thoughts, voices, and emotions, can lead to negative feelings. **Establishing and finding strategies**: establishing workable and helpful strategies, rediscovering strategies from the past. **Homework:** Listening to recordings of sessions, keeping an ACT diary to document useful things and note topics and questions for the next session, repeating exercises using a therapy manual and mindfulness CD.	1-15 sessions in a 1–2-week rhythm, additional homework Experienced clinical psychologists Specific training on ACT as well as supervision by ACT experts (no details on exact content and scope)	**Quantitative:** No specific results reported (13)^a^ **Qualitative:** ACT can help reduce intensity and distress of voices. Intervention was experienced as very helpful by participants and recommended to others (3)^a^
**iMPV** Individual mindfulness Programme for Voices	1 37 14	**Basics:** iMPV is based on mindfulness based cognitive training (MBCT) and mindfulness based stress reduction (MBSR). Adaptations were made for people who hear voices. Mindfulness exercises are time limited to 15 min and are done with open eyes. **Content of sessions:** demonstration and guidance of mindfulness exercises; conversations and exercises to replace habitual responses with mindful ones; establishing non-judgmental awareness and acceptance of voices; self-recorded audio recordings of own imitated voices were used when voices were not present in the exercise sequences. **Homework:** homework exercises are given as well as a protocol to be completed.	4 sessions over 4 weeks and additional homework Professionals with psychosis-specific CBT and ACT training Specific MBCT training (no information on content and length of training and supervision).	**Quantitative:** *PSYRATS-AH, total:* No significant improvement (37)^a^ Effect size low: 0.24 (37)^a^ *Life impairment (item, PSYRATS-AH)*: significant improvement (37)^a^ Effect size: medium: 0.43 (37)^a^ **Qualitative:** Study participants recommend the intervention to other people who hear voices. Intervention helps to calm down despite negative voices and to focus on positive things and well-being (37).^a^
**EFC/ MsV** Experience Focused Counselling with voice hearers and talking with voices	6 51, 52^b^, 53^b^, 54, 58, 59 29^c^	**Four-part intervention**. Randomised case series (58) with stronger focus on the 4th part—talking with voices. **Basic assumptions:** hearing voices is not a symptom of a disease, but a normal, human experience that can be understood within the context of life events. **Maastricht Interview (semi-structured)**: open conversation about the voices and experiences, focus on subjective meaning and context of the experience **Maastricht Report:** written report of the contents of the interview in the tongue of the voice-hearing person **Maastricht Construct:** joint development (voice hearer and accompanying person) of subjective and meaningful explanatory model regarding the voice-hearing experience within the voice hearer's life context **Talking with voices:** indirect or direct dialogue by the accompanying or the voice hearing person with the voices with the aim to give space to what the voices want to say	18–40 sessions over 9–10 months Clinical psychologists (58) or health professionals (52) from the fields of nursing, psychology, pedagogy, or social work 6-day EFC training and an extra 2 days on talking with voices in the case series Regular group and individual supervision over the training and intervention phase	**Quantitative:** *PSYRATS-AH, total:* Significant improvements (58)^a^ Effect sizes: medium to large 0.76–1.57 (58)^a^ Effect sizes: small to large 0.4–1.0 (52)^a^ *Other voice hearing measurements (BAVQ-R, DAIMON):* Significant improvements (58) ^a^ Effect sizes: small to medium −022–0.78 (58)^a^ **Qualitative:** Positive evaluation of EFC/ MsV by voice hearers and mental health professionals. Easy to implement and helpful to understand voices in the context of life and their meaning. Correlation between subjectively assessed improvement and better understanding and more control over voices. Talking with the voices, though not negative, was not experienced positively in all cases (51,53,59)^a^
**Relating therapy** Therapy to reflect and improve on the relationship with the voices and the environment	2 24, 25 34	**Three-part intervention:** **Basic assumptions:** Parallels between the way that voices are related to and how people are related to in interpersonal relationships. Use of role plays as a method to practice assertive relating to the voices. **1st phase:** getting to know the intervention and its impact on the relationship between the person and their voices. **2nd Phase:** exploring themes in the voice-hearing person's history of relating in the relationships with the voices as well as within interpersonal relationships. **3rd Phase**: exploration and development of assertive communication with the voices and people in the social environment.	12–16 sessions over 3–4 months Therapists from psychology and nursing backgrounds with a lot of experience with people who hear voices Specific training (unspecified), no information on supervision, to check therapy compliance a checklist with information on the different phases of the intervention has to be used	**Quantitative:** Effect sizes: large 1.2–1.4 (25)^a^
**PE und VRE** Prolonged Exposure and Virtual Reality Exposure Therapy	1 14 162	**Preparation:** Explaining basic principles of treatment and psychoeducation to understand trauma, breathing training and other interventions in preparation for exposure. **After the 3rd session**: start of exposure therapy. **PE:** Therapist guided imagination of past trauma events, creating a sense of safety, goal of being able to do avoided exposures again in real life. **VRE:** Same as PE. Instead of imagination, virtual reality. VE was created with a computer programme; participants see and move around in it with virtual reality glasses.	10 sessions of 90–120 min each doctoral level psychologists 2-day specific training, regular supervision	**Quantitative:** No significant improvement Reduction in the number of participants who reported hearing voices at all from 39.1 to 26.8%
**EMDR** Eye Movement Desensitization and Reprocessing	1 39 36	**Basic assumptions**: EMDR was developed for PTSD. Large overlap of negatively perceived emotions in intrusions in PTSD as well as in the occurrence of voices. EMDR uses the limited capacity of the working memory. Simultaneous tasks such as holding emotional memory in memory and making eye movements (visual appraisal, VT) or counting aloud (auditory appraisal, AT) decreases emotionality of memory. **Therapy session:** Within one session, the different appraisal methods (VT, AT) are performed over five different sequences, alternating for 5 min each.	One session, alternating between VT and AT and control intervention in different sequences, 2 times each for 5 minutes. Not reported Not reported	**Quantitative:** No hearing voices outcome reported *Subjective assessment of discomfort (SUD) when remembering hearing voices*: Significant improvement

a Numbers related to Supplementary Table S1.

b Studies in which nurses are named as interventionist.

c Total of participants in the study, some related in the same time to the quantitative, and qualitative part of the study.

#### CBTp

CBTp with a clear reference to voice hearing was the subject of one systematic review, two meta-analyses and 15 additional individual studies which had not been included in the systematic review or the meta-analyses. The systematic review [36] and the two meta-analyses [15, 21] included a total of 20 individual studies and 987 participants (studies included in more than one review were only counted once). The remaining 15 individual studies [11, 17, 28, 29, 31, 33, 35, 40, 41, 43, 56, 61, 68, 73, 74] included 945 study participants. The CBTp approaches generally included relationship building and normalization, psychoeducation, a case formulation and the defining of individual goals at the beginning of the process. This would then be followed by working on improving the individual's handling of stress, as stress is attributed as causal to the voices, and the development of coping strategies. Some adaptations have seen CAT [74] complement therapy with a stronger emphasis on managing everyday life situations; the use of specific techniques for dissociative symptoms in the context of PTSD [73] and apply trauma specific exposure components in people diagnosed with psychosis and PTSD [29]. Interventionists were mostly comprised of psychologists with a master's degree or doctorate. They had normally received specific further training and had had some years of professional experience as therapists, though it was not always clear in which specific fields. In six studies other professionals, such as psychiatrists, occupational therapists and nurses were named as interventionists [17, 21, 28, 31, 40, 41, 43]. In most cases, specific training for professionals was described, ranging from 10 h [35] to a 5-day course [68] to 120 h of multimodal training [28]. Interventionists would normally be supported with individual supervision at a frequency of weekly to fortnightly. There was a range of roughly 5–25 therapy sessions over a period of 1–9 months. One exploratory observational study [35] found a minimum “dose” of 15 CBTp sessions for a statistically significant reduction and 25 sessions for a maximum reduction in positive symptoms. Ten studies showed statistically significant improvements in voice hearing at the end of the intervention or at a follow-up time point [28, 29, 33, 40, 41, 43, 61, 73, 74], two studies did not [56, 68] or only on one dimension of a scale [31]. The effect sizes ranged from small to large. In the one qualitative study [11], more control and power over the voices and a normalization of the voice experience were reported but fear of responding to the voices remained.

#### AVATAR therapy

One systematic review and meta-analysis [1] involving 3 individual studies and 195 study participants and an additional individual study [60] involving 23 study participants were identified. AVATAR therapy is a computer assisted approach based on the cognitive model of auditory hallucinations (41). In this process, software is used to create a computerized voice and image of the main voice heard by the voice hearing person. The interventionist sits in another room and supports the voice hearing person in asserting themselves toward the voice. The interventionist can interact with the voice hearing person directly or *via* the computerized voice. In a modified approach [60], the interventionist supported the voice hearing person in the same room. In addition to practicing a confident response to the voice, a next step is to have experiences with the computerized voice and image which aim to move communication toward a more constructive and supportive direction. AVATAR therapy has so far been conducted by clinical psychotherapists (42) with experience in psychological therapies such as CBTp. The interventionists were guided by a manual and team discussions. There was no indication of the length and content of the training though weekly supervision sessions by an experienced therapist were described. AVATAR therapy was implemented over a period of about 2 months in 6–7 weekly sessions. There were statistically significant improvements in both studies on at least one voice hearing measurement time point (post-therapy or follow-up).

#### MsV aka EFC

MsV *aka* EFC (EFC is the name used for MsV in the German speaking countries) was investigated in five individual studies [51–53, 58, 59] and one systematic review [54]. Two individual studies were qualitative evaluations of a pilot RCT on EFC [51, 53] and one was a qualitative investigation of a randomized case series of MsV [59]. The systematic review published in 2014 included no published studies involving all elements of EFC/MsV. Since then, the two randomized studies [52, 58] included a total of 27 study participants. MsV/EFC assumes that voice hearing is a normal human perceptual variation which should be understood within the context of life events. It generally uses a three stage process (34), which consists of: the semi-structured Maastricht Interview as an open exploration of the subjective voice hearing experience; a written report summarizes the contents of the interview from the perspective of the person, which is then used to develop a subjective, meaningful explanatory model (the construct) in the third step. Talking with voices can be used as an explorative approach alongside these three parts to get to know the voices and their messages better. In the case series [58], clinical psychologists with general clinical experience engaged in MsV/EFC. In the pilot RCT [52] the interventionists had various professional backgrounds, including nursing. All of them received a 6-day training on EFC and, in the more recent study [58], an additional 2 days on talking with voices. The training was conducted by experienced experts in MsV/EFC and talking with voices. Regular individual and group supervision were described. In the first study [52] the approach was offered in 2–3 weekly sessions which amounted to an average of 10 min/week over 10 months. In the more recent study [58] 18–20 appointments were offered over a period of 9 months. There were large effect sizes in relation to voice hearing outcomes at the end of both studies (*d* = 0.76/1.0) and at 3-month follow up in the recent-study (*d* = 1.57). In the qualitative evaluations both professionals and voice hearers [51, 53, 59] felt MsV/EFC to be a positive and easy to implement approach, that had been largely helpful in improving constructive ways of dealing with voices related distress. Although two participants did not experience the talking with voices part positively but a frightening and hard thing to do, they did feel it to be powerful at the same time.

#### Relating Therapy

Two individual quantitative studies [24, 25] were available on Relating Therapy, involving a total of 34 study participants. Relating Therapy emphasizes a need for assertive communication when relating to voices and people and assumes a reciprocal character between these relationships. After an initial time of rapport building past relationships of the voice hearer with people are explored to notice relating patterns. The assumption here is that the ways voice hearers relate to other people might well be similar to the way they relate to their voices, too. This is followed by a focus on the relationship with those voices that are described as abusive, critical, or bullying. Voice hearers are then encouraged by their interventionist to engage in role playing (i.e., imagining that they are relating to the voice), in order to improve their levels of assertiveness toward the voices. There is no exploration of content or meaning of the voices (43). Relating Therapy was conducted by interventionists from psychology and nursing backgrounds with a lot of experience in working with people who hear voices. They received specific training, but no information on the content, scope, or nature of supervision was provided in the study articles. A random selection of recorded sessions was checked for therapy compliance by an independent rater filling out a related checklist. Therapy was carried out in 12–16 weekly sessions over 3–4 months. In the first study [24], a reduction in distress and/or an improvement in the controllability of voices was achieved in four of the five cases examined. In the pilot RCT [25] large effect sizes were found at 16 weeks (*d* = 1.4) and 36 weeks (*d* = 1.2) using measures of voice hearing distress.

#### Acceptance and Commitment Therapy

There was one qualitative study [3] and one systematic review with meta-analysis [13] on ACT, with the latter including eight individual studies, seven of which on ACT in individual settings, totaling 274 individual study participants. In ACT therapy begins with the introduction of the ACT approach. During the implementation, cognitive defusing takes on a central role. In this process, the interventionist may take over and play the role and activities of the voices or thoughts. The voice hearer observes whilst trying not to evaluate their own feelings, voices, or thoughts, and without communicating with the “voices or thoughts” or implementing recommendations. In this way, the voice hearer can experience that without controlling and suppressing negatively felt thoughts, voices and emotions, negative feelings or discomfort can in fact be reduced. In a next step, implementable and helpful strategies are identified and established. Homework includes listening to recordings of the sessions, keeping an ACT diary and noting topics and questions for the next session. ACT was delivered by experienced clinical psychologists with specific training and supervision provided by ACT experts. No details of the exact content and scope of the training were given. ACT comprised between 1 and 15 sessions of 45–60 min at weekly or fortnightly intervals. The meta-analysis of the review [13] reported no voice-specific outcomes. However, the impact on positive symptoms, found small effect sizes (*g* = 0.21). The results of the qualitative study [3] with 9 participants of an RCT (44) included in the meta-analysis, suggested that ACT can help reduce the intensity and distress of voices. Overall, ACT was experienced as helpful by the participants and recommended to others.

#### Smartphone-based Coping-focused Intervention for voice hearing (smartphone CFI)

One study [9] reported on smartphone CFI for voice hearing. A pilot RCT on the effectiveness of the approach included 34 participants. Coping Strategy Enhancement (CSE) (45), a key element of CBT based therapy, formed the primary basis of the approach. In essence, the voice hearer is encouraged to continue to use strategies that have been assessed as useful and effective in the past. The focus of the first session comprises an introduction to the approach. It also includes training on how to use the smartphone app and how to collect data on voices and corresponding strategies. After a subsequent 6-day monitoring phase of data on voices and strategies and their respective evaluation, alternative strategies used by the voice hearer in the past for dealing with voices are identified and defined in a second session. In a subsequent 10-day period, participants receive regular prompts to apply the strategies. In addition, corresponding data continues to be collected on a regular basis. Completion was achieved after a further analysis and intervention round as described before. For the qualification, training and supervision of the professionals, reference is made to a single case study illustration (46), which indicates that the approach in the included study was seemingly conducted by the same PhD psychologist named in the feasibility study. No reference was made to prior working experience. The four counseling sessions took place over 1 month. A statistically non-significant improvement with a medium effect size (*g* = 0.55) was reported for voice hearing distress. However, subjective assessment of coping with the voices and understanding of the voices showed significant improvements with high (*g* = 1.45) and medium effect sizes (*g* = 0.61).

#### Prolonged and Virtual Reality Exposure Therapy

As part of a secondary analysis [14] of an RCT with a wait list design by Reger et al. (47), one study investigated the combination of two different forms of exposure therapy for post-traumatic stress disorder (PE; VRE) in participants with PTSD and psychotic-like experiences (PLE), which included “persecutory ideation” and “auditory or visual hallucinations”. The approach started with a psychoeducational introduction to treatment and trauma understanding and included breathing training and training to normalize responses to traumatic events in preparation for exposure. After the 3rd session, exposure therapy started. PE included therapist guided imagination of past trauma events and exposure to cognitive and emotional processes and experience. This was accompanied by work on a sense of safety to reduce the avoidance of real-life exposures. VRE took place in the same way as PE except that a computer based virtual reality was used instead of imagination. All interventionists were doctoral level clinicians trained in clinical psychology. They had also received a 2-day workshop on PE and VRE. In addition, they carried out at least two virtual treatments under supervision as preparation and received regular supervision by particularly experienced therapists. The therapy lasted 10 sessions of 90–120 min each. Regarding the time by treatment (PE or VRE vs. waitlist) effect related to voice hearing, non-significant improvements were reported without indication of effect sizes. There was a reduction in the number of participants from 39.1 to 26.8% who reported hearing voices at all.

#### Eye Movement Desensitization and Reprocessing

One study on EMDR [39] was identified. This was an intervention study with 36 participants who were described as suffering from auditory hallucinations and had been mainly diagnosed with a psychotic disorder. A within-subject design to examine two different EMDR approaches (visual and auditory taxation) vs. a control intervention was used. The EMDR protocol developed for PTSD was used for people with voice hearing distress as it was argued that negative emotional memories play a similar role in intrusions in PTSD as in voice hearing. The goal of EMDR was described to decrease the emotionality of auditory memories of auditory hallucinations. In the intervention, participants perform two tasks alternately—emotional recall or remembering voices and performing eye movements (visual taxation, VT) or counting aloud (auditory taxation, AT). EMDR hypothesizes that it uses the limited capacity of working memory. By simultaneously moving the eyes (VT) or counting (AT) as well as keeping an emotional voice in the memory, emotionality decreases. No information was provided on the level of qualification, training, and supervision of the interventionists. All participants underwent a session in which the two different approaches as well as the control intervention were alternated 2 times for 5 min. Voice hearing distress was measured *via* a short subjective assessment of the degree of discomfort (subjective units of disturbance, SUD) in relation to the memory of voice hearing. This was assessed by the study participants before and after each approach sequences. The results showed that the combined implementation of the two approaches (VT & AT) was significantly superior to control.

#### Individual Mindfulness-based Program for voice hearing

iMPV was investigated in a non-randomized pilot study [37] with 14 participants. iMPV is based on approaches from mindfulness-based cognitive therapy (MBCT) and mindfulness-based stress reduction (MBSR) and was adapted to people who hear voices. Adaptations included limiting the time of the mindfulness exercises to a maximum of 15 min and not closing the eyes. In the sessions, mindfulness exercises were guided, and people practiced replacing habitual reactions to voices with mindful reactions and acceptance with the aim to establish these as their new way of relating to the voices. Audio recordings of voices which had previously been imitated by the voice hearer were used when voices were not present in the practice sequences. Homework included exercises and keeping a log. The interventionists conducting iMPV in the study were not defined in terms of profession. They had training in CT and ACT for psychosis and received specific training in MBCT (length of training and supervision were not specified). iMPV included 4 weekly sessions and homework to be completed between sessions. Results showed no statistically significant improvement in terms of voices (PSYRATS-AH) with a low effect size (*g* = 0.24). Analysis of the disruption to life item from the PSYRATS-AH showed a statistically significant reduction at medium effect size (*g* = 0.43). In the qualitative evaluation, all study participants stated that they would recommend the approach to other people who hear voices as well as they would continue with the learned mindfulness practice. The materials were assessed as supportive, or the practices were felt to have been helpful to calm down despite the negative voices or in shifting attention from problems and difficulties to a sense of wellbeing.

## Discussion

This review included 77 articles on talk-based approaches for people who are distressed by their experience of hearing voices. In just over half of these, nine different approaches were identified which are explicitly aimed at people who hear voices. From these, most of the studies had been done on CBTp. However, newer developments in the tradition of CBT, such as AVATAR therapy and Relating Therapy, have also included a relational stance (42, 48) ACT and smartphone-based CFI, as well as trauma-focused approaches, such as EMDR or PE & VE, were also identified. In addition, studies on MsV/EFC, the individual approach associated with the Hearing Voices Movement, were also included. The various approaches differed greatly in terms of the number of sessions and the length of time they were offered. Most of the approaches were carried out by masters or doctoral degree psychologist. In some studies of CBTp, Relating Therapy and in MsV/EFC other health professionals, including nurses, were named as interventionists. Approach specific training and supervision for the interventionists were mostly described. When training and supervision was quantified a wide time range was identified. Most of the approaches showed positive outcomes in relation to voice related distress levels, though there was a wide range of effect sizes from small to large. The ACT and EMDR included studies did not use or report voice-specific outcome measures.

The results showed that the various approaches are at very different stages of development. For example, iMPV [37], MsV/EFC [52, 58], smartphone-based CFI [9], or Relating Therapy [24, 25] have only recently started with piloting or conducting fist small, randomized trials and are not yet in a position to provide generalizing results. The trauma focused approaches EMDR [39] and PE and VRE [14], too, have only recently started to apply their methods to voice hearing in some small studies. AVATAR therapy and ACT have shown the strongest development since the overview of Thomas et al. (8). For both a systematic review and meta-analyses do now exist [1, 13] meaning there is now more evidence toward generalizing their findings. However, the small number of included studies (e.g., for AVATAR therapy only 3 studies were included in the review [1]), shows that more research is needed even for these approaches and the current results should be considered with caution. There have also been some developments in the MsV/EFC approach since its last review [54], with early indications showing promising results, too. As many of these approaches have only recently been developed or started to be studied using more traditional research designs it remains to be seen how helpful, effective, and applicable they will be for people who are distressed by their experience of hearing voices. All of them may well turn out to have some benefits and thus complement each other and provide greater client choice (43).

Most of the identified approaches belong to the tradition of using a behavioral and cognitive approach, and here particularly to the second and third wave of development which started to focus more on the respective subjective experiences of users. CBTp or voice specific CBT approaches, and smartphone-based CFI can be referred to as belonging more to the second wave of CBT approaches. These are characterized by the assumption that people's life problems were mainly due to disturbances in perception, thinking and behavior, as a symptom of a mental illness. Therapy would thus aim to address and improve on these areas (49). Rather than trying to change the form, frequency, or situational sensitivity of so-called “negative” or “pathological” emotions or thoughts, third wave approaches focus also on the function of cognitions and emotions in the context of the social environment and severe life events and put more attention on the person's relationship to his or her own experience (49). These third wave developments included ACT, mindfulness based approaches as well as AVATAR and Relating Therapy may be considered more in line with calls for “alternative” approaches to therapy and support for people who hear voices and are distressed by the experience. This is because they appear to be more open to considering the subjective views and explanatory models of voice-hearers (50), as well as the relationship of individuals to themselves, to the voices and to people in the social environment (51).

The MsV/EFC approach (52), though it first appeared in 1987, has largely developed as part of a user-led civil rights focused Hearing Voices Movement. As such, it has naturally incorporated many elements found in the other approaches, such as the importance of a trauma focus, assertiveness, and a change in relating to the voice hearing experience. As there has always been a philosophy of learning from voice hearers and voices directly this has also meant that it has gone a few steps further. The “disease” reference to voice hearing is completely rejected and replaced with the understanding of voices as a normal human experience and subjectively sense making reactions within the person's life context, which regularly contains severe life events and/or trauma (53). Consequently, the focus is no longer on actively trying to correct “negative” or “aberrant” emotions, thoughts, behavior, or relations. Instead, voices, emotions, thoughts, behaviors and even ways of relating are acknowledged as understandable, normal, personal, subjectively meaningful experiences. Learning about the voices' potential meaning and roles in the individual's life can thus also be facilitated through the MsV/EFC process and if so, desired also as part of an active consultation/ talking process with the voices. The voices are encountered as offering valuable information, advice, or insights for an improvement in the person's life experience (54).

The increased number of talking approaches to support people who are distressed by the experience of hearing voices fits well with the on-going discourse around the plurality of different theories, conceptualizations, and explanations of mental and emotional distress. In a review of different explanatory models of mental illness, diseases, and disorders Richter and Dixon (55) identified 34 different theories that are assigned to the five overarching categories: Biology, Psychology, Social, Consumer, and Cultural. They argue that mental health services should consider and include the varied different conceptualizations and explanatory models much more in the provision of services than is currently widely the case. This applies particularly to considering and then providing services in line with the preferences and understandings of the respective service user. This might mean providing the MsV/EFC approach or a more cognitive approach. It might also make sense to use an eclectic approach (3), which appears to be in line with what some practitioners, for example in the UK (56), already prefer to provide in practice anyway.

The kind of approach specific training and supervision needed as well as a strong focus in some countries on professionals needing to possess formal psychotherapeutic training before they are allowed to work therapeutically with clients represents, among others, an existing barrier for people with mental health needs in accessing helpful or therapeutic approaches in some services in the German speaking part of Europe (57, 58), the UK (18) and the US (59). This review identified some differences in backgrounds of the professionals conducting the approaches. In most approaches, e.g., CBTp, smartphone-based CFI, ACT, PE and VRE as well as in one of the MsV/EFC studies, psychologists with a master's degree or doctorate were usually mentioned as interventionists. In some studies, such as in the pilot EFC study [52], the cognitive nursing interventions study [17], in Relating Therapy [25] and in some CBTp studies [31, 43, 56, 73] nurses and some other non-psychotherapy professions were also applying the approaches. Expanding on the professional backgrounds able to provide these approaches is also in line with the recommendations by Thomas et al. (8), who make a compelling case for greater delivery of novel talking approaches within routine service contexts, thus going beyond the traditional model of consultation room delivery. This would particularly require nurses, the biggest professional group in mental health services (60) and other “frontline” or “on the floor” staff, which form and play a key role in the implementation of such approaches (61), to be included in the provision of these approaches. Frontline staff, such as nurses, will normally have the chance to build great competencies for a constructive way of relating in everyday and often highly emotional relationship situations. Thus, they would potentially be very well suited to accompany voice hearers in what can at times be an emotional process. Fittingly, recent discussions and guideline recommendations have asked for nurses to be carrying out therapeutic approaches in Europe, such as in the UK (1) and in the German speaking part (62, 63), even though regulatory questions persist. However, within a dominant biomedical model in practice, also promoted by various psychiatric professional bodies and drug companies, the implementation of newer, often paradigm challenging approaches, has to contend at the very least with well-known resistances to change.

### Limitations

To our knowledge, this is the first review of all talk-based individual approaches for people distressed by their experience of hearing voices that includes a trans professional, trans diagnostic, trans methodology and trans approach focus. Both the nature of a scoping review design and the respective varied stages of approach specific development make a head-to-head comparison of the relative effectiveness and contribution of the approaches difficult at this stage. In particular, the fact that we did not extract data or results from primary studies, which were part of included reviews, may have led to a less detailed description of the effectiveness of approaches or interventions, for example, in relation to outcomes. Also, the descriptions and contents of the respective approaches were synthesized from the identified studies. This may have led to some reductionist representations and reflections of the approaches in relation to primary literature. So, consultation of primary literature to get a more in-depth description of the respective approach is required. As this review focused on individually implementable approaches it also does not account for other important developments in recent decades, such as group or team-based approaches, for example, Hearing Voices Groups or Open Dialogue, which are already being implemented in some services.

## Conclusion and implications for further research and clinical practice

It seems that all the talk-based approaches identified in this scoping review show some promise of positive effects for voice hearers who are distressed by their experience of hearing voices. Although some are further in their development of a convincing evidence base than others, all would benefit from more focused research. For most this would mean the inclusion of qualitative studies to better understand how different voice hearers relate to the differing approaches on offer. As none of the approaches identified overall or voice specific deteriorations there appears to be a strong case for the implementation of all of these approaches in practice. A greater emphasis on whole systems implementation and thus in the provision of these approaches by the inclusion of frontline staff, such as nurses, pedagogues, social workers, occupational therapists, etc. would seem to be helpful. Far from being a disadvantage, the heterogeneity of approaches would seem to better suit a recovery focused client led service than is currently often the case. This would also be in line with recommendations and requirements by people who are distressed by their voice hearing experience. Health professionals or service users should use these findings to discuss a broader understanding of hearing voices in their services and the implementation of alternative forms of accompaniment, support and therapy for people who hear voices. An implementation of these approaches will no doubt necessitate less financial and staff cuts than has become common practice. It would also require further training, but also the development of a greater openness toward other forms of thinking and in many cases a preparedness to work with the dynamics of paradigm shifts. This will regularly necessitate a readiness by all stakeholders to continue to work on changing attitudes. The resulting anecdotally evident greater satisfaction for all stakeholders may also contribute to a greater ability for service providers to retain staff in times of known recruitment difficulties in several countries.

Given our current understanding of the trans diagnostic nature of hearing voices it would also make sense to include more trans diagnostic and trans professional research which includes nurses as the biggest professional group and thus available resource, in future research. Finally, a transdiagnostic systematic review, meta-analyses and -synthesis of the range of approaches introduced in this scoping review, once a greater quantity of high-quality studies exists for all of these approaches, would allow for greater comparability.

## Data availability statement

The original contributions presented in the study are included in the article/Supplementary material, further inquiries can be directed to the corresponding author.

## Author contributions

CB conceptualized and designed the study, contributed to all of the steps of the study selection process, conducted data extraction and synthesis, and wrote the first draft of the manuscript. JS reviewed the search strategy and in-/exclusion criteria, contributed to all of the steps of the study selection process, and reviewed the data extraction. FW reviewed the conceptualization and design of the study. All authors contributed to manuscript revision, read, and approved the submitted version.

## Conflict of interest

The authors declare that the research was conducted in the absence of any commercial or financial relationships that could be construed as a potential conflict of interest.

## Publisher's note

All claims expressed in this article are solely those of the authors and do not necessarily represent those of their affiliated organizations, or those of the publisher, the editors and the reviewers. Any product that may be evaluated in this article, or claim that may be made by its manufacturer, is not guaranteed or endorsed by the publisher.
