# Supplementary material for: Talk-based approaches to support people who are distressed by their experience of hearing voices: A scoping review
Source: Front Psychiatry. 2022 Oct 10;13:983999. doi: 10.3389/fpsyt.2022.983999 (PMC9589913; doi:10.3389/fpsyt.2022.983999)
Supplement: Supplementary file 1 [file Data_Sheet_1.docx]

Supplementary Material

# Supplementary Figures and Tables

**Supplement table 1:** Analysis of all included studies in the review (n = 77)

| **Study** | | |  |  | **Intervention** | | | | | | | | | **Methods / Design^a^** | | | | | | **Outcome measures^a^** | | | | | | **Results^a^** | | | | | | | | | |
| --- | --- | --- | --- | --- | --- | --- | --- | --- | --- | --- | --- | --- | --- | --- | --- | --- | --- | --- | --- | --- | --- | --- | --- | --- | --- | --- | --- | --- | --- | --- | --- | --- | --- | --- | --- |
| **Nr.** | **Identifier** | **Country^a^**  (ISO-Code) | **Number of included studies in Reviews (N)** | **Number of included participants (n)** | **Diagnosis, phenomenon or symptom focus** | | | | | | **Name^a^** | **Number of sessions**  **(over X months)** | **Control condition^a^** | **Quant.** | | | | **Qual.** | | **PSYRATS** | **PANSS** | **SAPS** | **BAVQ-R** | **BPRS (-E)** | **Others^b^** | **Quantitative results:**  ***Relevant outcomes:*** if no total scores available subscales or items were used if indicated; positive symptoms (e.g., PANSS-, & BPRS-E positive, PSYRATS-Total); hearing voices or auditory hallucinations (e.g., PSYRATS-AH, BPRSE-Hallucination Item)  ***Statistically significant improvements (p-value < .5; sig.)*:** yes (+), no (-), or not available (N/A)  **Effect size (ES)**: value (standardized mean differences, Cohen’s d, or Hedge’s g) or not available (N/A)  **pre-, to post-test (t0-t1) / pre-test to follow-up (t0-t2):** 4 cells per row  **Group x X-timepoints:** 2 cells per row  **Qualitative Results** are described in one row | | | | | | | | |  |
|  |  |  |  |  | **Psychosis** | **Schizophrenia** | **Voices / auditory hallucinations** | **Delusions** | **Trauma** | **Others** |  |  |  | **Systematic Review** | **Meta-Analyses** | **RCT** | **Others** | **Single Study** | **Qualitative- or Meta-Synthesis** |  |  |  |  |  |  |  |  |  |  |  |  |  |  |  |  |
|  |  |  |  |  |  |  |  |  |  |  |  |  |  |  |  |  |  |  |  |  |  |  |  |  |  | **Outcome** | **sig.**  **t0-t1** | | | **sig.**  **t0-t2** | **ES**  **t0-t1** | | **ES**  **t0-t2** | |  |
|  |  |  |  |  |  |  |  |  |  |  |  |  |  |  |  |  |  |  |  |  |  |  |  |  |  |  | **sig. group x**  **X-timepoints** | | | | **ES group x**  **X-timepoints** | | | |  |
| 1 | Aali et al. (2020) | GBR, CAN | 3 | 195 | x | x | x |  |  |  | AVATAR therapy | 6- 7 (2) | AC or TAU | x | x |  |  |  |  | x | x | x | x |  |  | **AVATAR vs TAU**  PANSS-positive: BAVQ-R: | -  + | | | -  N/A | N/A  N/A | | N/A  N/A | |  |
|  |  |  |  |  |  |  |  |  |  |  |  |  |  |  |  |  |  |  |  |  |  |  |  |  |  | **AVATAR vs AC**  PSYRATS-AH: BAVQ-R: | +  + | | | -  N/A | N/A  N/A | | N/A  N/A | |  |
| 2 | Adams et al. (2020) | NLD | 6 | 236 | x | x |  |  | x |  | EMDR | 1-10 (N/A) | PE & TAU | x |  |  |  |  |  | x | x |  |  |  |  | **EMDR vs TAU:**  PSYRATS-AH: | + | | | - | N/A | | N/A | |  |
|  |  |  |  |  |  |  |  |  |  |  |  |  |  |  |  |  |  |  |  |  |  |  |  |  |  | **EMDR (within group):**  PSYRATS-AH: | + | | | N/A | N/A | | N/A | |  |
| 3 | Bacon et al. (2013) | AUS |  | 9 | x | x | x | x |  |  | ACT | 8 (N/A) |  |  |  |  |  | x |  |  |  |  |  |  |  | Reduction of the intensity and distress of the voices, more control over the voices | | | | | | | | |  |
| 4 | Balzan et al. (2014) | AUS |  | 28 |  | x |  |  |  |  | MCT | N/A | TAU |  |  |  | OS |  |  |  | x | x |  |  |  | PANNS-positive: | | + | - | | | N/A | | N/A |  |
| 5 | Balzan et al. (2019) | AUS |  | 54 |  | x |  | x |  |  | MCT | 8 (N/A) | AC |  |  | x |  |  |  |  | x |  |  |  |  | PANNS-positive (within group): | | + | + | | | 0.83 | | 0.29 |  |
|  |  |  |  |  |  |  |  |  |  |  |  |  |  |  |  |  |  |  |  |  |  |  |  |  |  | PANNS-positive (group x time): | | + | | | | N/A | | |  |
| 6^c^ | Barrowclough et al. (2010) | GBR |  | 327 |  | x |  |  |  | Addiction | CBT & MI | 26 (12) | TAU |  |  | x |  |  |  |  | x |  |  |  |  | PANNS-positive: | | - | - | | | N/A | | N/A |  |
| 7 | Barrowclough et al. (2014) | GBR |  | 110 | x |  |  |  |  | THC | CBT & MI brief or  CBT & MI long | 12 (4.5)/  24 (9) | TAU |  |  | x |  |  |  |  | x |  |  |  |  | Long vs. brief vs. TAU:  PANNS-positive: | - | | | - | N/A | | N/A | |  |
| 8 | Beames et al. (2020) | AUS |  | 16 |  | x |  |  |  |  | CBT | 20 (N/A) | CBT group |  |  |  | MS |  |  | x |  |  |  |  |  | PSYRATS-AH: | - | | | N/A | N/A | | N/A | |  |
|  |  |  |  |  |  |  |  |  |  |  |  |  |  |  |  |  |  |  |  |  |  |  |  |  |  | Participation in the individual intervention was more reliable | | | | | | | | |  |
| 9 | Bell et al. 2020 | AUS |  | 34 |  |  | x |  |  |  | Smartphone-based CFT | N/A | TAU |  |  | x |  |  |  | x |  |  |  |  | a | PSYRATS-AH:  Coping with voices (VAS):  Understanding Voices (VAS): | -  +  + | | | N/A  N/A  N/A | 0.5  1.4  0.6 | | N/A  N/A  N/A | |  |
| 10 | Berry & Hayward (2011) | GBR | 8 | 81 | x |  |  |  |  |  | CBTp (individual & group) | N/A |  |  |  |  |  |  | x |  |  |  |  |  |  | Few statements about voices. Improved dealing with voices and a feeling of more power over the voices. | | | | | | | | |  |
| 11 | Birchwood et al. (2018 | GBR |  | 25 |  |  | x |  |  |  | CBTp (CTCH) | N/A |  |  |  |  |  | x |  |  |  |  |  |  |  | More control and power over the voices, normalisation of the voice experience. Fear of responding to the voices and of contradicting them. | | | | | | | | |  |
| 12 | Brand et al. (2018) | AUS | 12 | 520 | x |  |  |  | x |  | Trauma-focused Interventions | 3-16 | TAU or AC | x | x |  |  |  |  | x | x |  |  | x |  | Positive symptoms:  Hallucinations: | +  - | | | -  - | 0.31  0.14 | | 0.18  0.06 | |  |
| 13 | Brown et al. (2020) | AUS | 8 | 274 | x |  | x | x |  |  | ACT (individual & group) | N/A | TAU or AC | x | x |  |  |  |  |  | x |  |  |  |  | Psychotic symptoms: | - | | | | 0.21 | | | |  |
|  |  |  |  |  |  |  |  |  |  |  |  |  |  |  |  |  |  |  |  |  |  |  |  |  |  | Notes: AC is superior to ACT; ACT is superior to TAU; group is superior to individual | | | | | | | | |  |
| 14 | Buck et al. (2019) | USA |  | 161 |  |  | x | x | x |  | PE & VRE | 10 | WL |  |  |  | CT |  |  |  |  |  |  |  | b | BASIS-24-Hallucinations: | - | | | N/A | N/A | | N/A | |  |
| 15 | Burns et al. (2014) | CAN | 12 | 639 | x | x | x | x |  |  | CBTp | 10-24 | TAU, WL or AC | x | x |  |  |  |  | x | x |  |  | x |  | Positive symptoms: | + | | | + | 0.47 | | 0.41 | |  |
| 16 | Drake et al. (2014) | GBR |  | 61 | x | x |  |  |  |  | CR before CBT | N/A | SC before CBT |  |  | x |  |  |  | x | x |  |  |  |  | PSYRATS-AH: | - | | | N/A | N/A | | N/A | |  |
|  |  |  |  |  |  |  |  |  |  |  |  |  |  |  |  |  |  |  |  |  |  |  |  |  |  | CR reduced number of CBT sessions | | | | | | | | |  |
| 17 ^c^ | England (2007) | CAN |  | 65 | x | x | x |  |  |  | CBT (CNI) | 12 (4) | TAU |  |  | x |  |  |  |  |  |  |  | x |  | Brief-BPRS-overall: | N/A | | | + | N/A | | 0.71 | |  |
| 18 | Farhall et al. 2009 | AUS |  | 94 | x | x |  |  |  |  | CBTp | 12-24 | TAU |  |  | x |  |  |  |  | x |  |  |  |  | PANSS-Positive: | - | | | N/A | N/A | | N/A | |  |
| 19 | Fichtenbauer et al. (2019) | AUT |  | 10 | x | x |  |  |  |  | DIALOG+ | 5 | - |  |  |  | OS |  |  |  | x |  |  |  |  | PANSS-Positive: | + | | | N/A | 0.77 | | N/A | |  |
| 20 | Garety et al. (2008) | GBR |  | 301 | x | x |  |  |  |  | CBT p & FI, CBTp | 20 | TAU |  |  | x |  |  |  |  | x | x |  |  |  | PANSS-Positive:  PSYRATS-AH: | -  - | | | -  - | N/A  N/A | | N/A  N/A | |  |
| 21 | Gould et al. (2001) | USA | 7 | 340 | x | x | x | x |  |  | CBTp | 5-20 (1-9) | TAU, WL, or AC | x | x |  |  |  |  |  |  |  |  |  |  | Positive symptoms: | + | | | + | 0.65 | | N/A | |  |
| 22 | Haddock et al. (2009) | GBR |  | 77 | x | x |  |  |  |  | CBTp | 25 | AC |  |  | x |  |  |  |  | x | x |  |  |  | PANSS-Positive:  PSYRATS-AH: | -  - | | | -  - | N/A  N/A | | N/A  N/A | |  |
| 23 | Harder et al. (2014) | DNK |  | 269 | x | x |  |  |  |  | SPP | (36) | TAU |  |  |  | CT |  |  |  | x |  |  |  |  | PANSS-Positive: | - | | | + | N/A | | N/A | |  |
| 24 | Hayward et al. (2009) | GBR |  | 5 |  |  | x |  |  |  | Relating Therapy | 12-24 | - |  |  |  | CS |  |  | x |  |  |  |  |  | Change in control and distress in four cases and in patterns of relating to voices | | | | | | | | |  |
| 25 | Hayward et al. (2017) | GBR |  | 29 |  |  | x |  |  |  | Relating Therapy | 16 (4) | TAU |  |  | x |  |  |  | x |  |  |  |  | c | PSYRATS-AH:  VAY 4 subscales:  - Voice dominance  - Voice intrusiveness  - hearer dependence  - Hearer distance | +  -  -  -  - | | | -  -  -  -  - | 1.4  0.4  0.1  0.5  0.7 | | 1.2  0.3  0.4  0.6  0.8 | |  |
| 26 | Husain et al. (2017) | PAK |  | 36 | x | x |  |  |  |  | CA-CBTp | 12 (3) | TAU |  |  | x |  |  |  | x | x |  |  |  |  | PSYRATS-AH:  PANSS-Positive: | +  + | | | -  + | 0.43  0.32 | | 0.42  0.25 | |  |
| 27 | Jacobsen et al. (2020) | GBR |  | 50 | x | x |  |  |  |  | MBCI | N/A | TAU or AC |  |  | x |  |  |  |  |  |  |  |  | de | HPSVQ:  Subjective Assessment of voices (number, burden, credibility): | N/A  N/A | | | -  - | N/A  N/A | | N/A  N/A | |  |
| 28 ^c^ | Jolley et al. (2015) | GBR |  | 20 | x |  | x | x |  |  | CBTp | 17 | - |  |  |  | OS |  |  | x |  |  |  |  |  | PSYRATS-AH: | + | | | N/A | 1.0 | | N/A | |  |
| 29 | Keen et al. (2017) | GBR |  | 9 |  |  | x | x | x |  | TF - CBTp | 25-66 (8-35) | - |  |  |  | CS |  |  | x |  |  |  |  |  | PSYRATS-AH: | + | | | - | N/A | | N/A | |  |
| 30 | Klingberg et al. (2012) | DEU |  | 198 | x | x |  | x |  |  | CBTp | 20 (9) | AC |  |  | x |  |  |  |  | x |  |  |  |  | Descriptive results: PANSS-Positive increase during intervention phase: No difference between CBTp and AC for 70% of participants | | | | | | | | |  |
| 31 ^c^ | Krakvik et al. (2013) | NOR |  | 45 |  |  | x | x |  |  | CBTp | 20 | WL |  |  | x |  |  |  | x |  |  |  | x |  | PSYRATS-AH subscales:  - physical  - emotional  - cognitive | -  -  - | | | -  +  - | 0.10  0.25  0.20 | | 0.10  0.15  0.10 | |  |

| 32 | Lamster et al. (2018) | DEU |  | 16 |  | x |  | x |  |  | CBTp | N/A | - |  |  |  | OS |  |  |  | | x |  |  |  |  | PANSS-Positive: | + | N/A | 1.7 | N/A |  |
| --- | --- | --- | --- | --- | --- | --- | --- | --- | --- | --- | --- | --- | --- | --- | --- | --- | --- | --- | --- | --- | --- | --- | --- | --- | --- | --- | --- | --- | --- | --- | --- | --- |
| 33 | Lewis et al. (2002) | GBR |  |  | x | x | x | x |  |  | CBTp | (1) | TAU & AC |  |  | x |  |  |  | x | | x |  |  |  |  | CBT-p vs AC  PANSS-Positive:  PSYRATS-AH: | -  + | N/A  N/A | N/A  N/A | N/A  N/A |  |
| 34 | Lincoln et al. (2012) | DEU |  | 80 | x |  |  |  |  |  | CBTp | N/A | WL |  |  | x |  |  |  |  | | x |  |  |  |  | PANSS-Positive | + | + | 0.61 | 0.65 |  |
| 35 | Lincoln et al. (2016) | DEU |  | 58 | x |  | x | x |  |  | CBTp | 5, 15, 25, 45 | - |  |  |  | OS |  |  |  | | x |  |  |  | f | CAPE-Positive (number and distress) | + | + | N/A | N/A |  |
| 36 | Lincoln & Peters (2019) | DEU | 8 | 588 | x | x | x |  |  |  | CBTp | 7-25 (2-9) | TAU or AC | x |  |  |  |  |  | x | |  |  | x |  | eg | PSYRATS-AH  (N = 3):  VCS (N = 2):  BAVQ-R (N = 1):  HPSVQ (N = 1): | N/A  N/A  N/A  N/A | N/A  N/A  N/A  N/A | 0.8-1.6  0.4 / 1.1  0.26  1.78 | 0.3-1.4  0.3 / 3.4  N/A  N/A |  |
| 37 | Louise et al. (2019) | AUS |  | 14 | x |  | x |  |  |  | MBCT | 4 (1) | - |  |  |  | OS |  |  | x | |  |  |  |  |  | PSYRATS-AH:  PSYRATS-AH - item life impact: | -  + | N/A  N/A | 0.24  0.43 | N/A  N/A |  |
| 38 | Ma et al. (2019) | HKG | 4 | 237 | x |  |  |  |  |  | CBFI | 7-16 (2-6) | TAU | x | x |  |  |  |  | x | | x |  |  |  |  | PANSS-Positive:  PSYRATS-AH: | +  - | N/A  N/A | N/A  N/A | N/A  N/A |  |
| 39 | Mathijsen et al. (2019) | NLD |  | 33 |  |  | x |  |  |  | EMDR (VT) | 1 | AC (AT & CC) |  |  |  | WS |  |  | x | |  |  | x |  | h | EMDR vs AC vs CC (within subject design) measuring SUD: | Superior effect of EMDR and AT reducing SUD compared to CC | | | |  |
| 40 | Morrison et al. (2004) | GBR |  | 87 | x | x | x | x |  |  | CBTp | 17 (7) | WL |  |  | x |  |  |  | x | | x |  |  |  |  | PANSS-Positive:  PSYRATS-AH subscales:  - physical  - emotional  - cognitive | +  +  +  + | +  -  -  + | N/A  N/A  N/A  N/A | N/A  N/A  N/A  N/A |  |
| 41 | Morrison et al. (2012) | GBR |  | 20 | x | x | x | x |  |  | CBTp | 26 (9) | - |  |  |  | OS |  |  | x | | x |  |  |  |  | PANSS-Positive:  PSYRATS-AH: | +  + | +  + | 0.87  056 | 1.08  0.70 |  |
| 42 ^c^ | Morrison et al. (2014) | GBR |  | 10 | x | x |  |  |  |  | MKT | 12 | - |  |  |  | OS |  |  | x | | x |  |  |  |  | PANSS-Positive:  PSYRATS-AH: | +  - | +  - | 1.24  0.54 | 1.27  0.47 |  |
| 43 ^c^ | Morrison et al. (2014b) | GBR |  | 74 | x | x | x | x |  |  | CBTp | 26 (9) | TAU |  |  | X |  |  |  | x | | x |  |  |  |  | PANSS-Positive:  PSYRATS-AH subscales:  - physical  - emotional  - cognitive | +  +  -  + | | 0.91  0.89  1.06  0.95 | |  |
| 44 ^c^ | Penn et al. (2011) | USA |  | 46 | x | x |  |  |  |  | CBTp (GRIP) | N/A | TAU |  |  | x |  |  |  |  | | x |  |  |  |  | PANSS-Positive (within group analyses): | + | + | 0.18 | 0.17 |  |
| 45 ^c^ | Peters et al. (2015) | GBR |  | 358 | x |  |  |  |  |  | CBTp | 9 (18) | WL |  |  |  | OS |  |  | x | |  |  |  |  |  | PSYRATS-AH: | + | + | 0.52 | 0.44 |  |
| 46 | Premkumar et al. (2015) | GBR |  | 60 | x |  |  |  |  |  | CBT | 19 (5) | TAU |  |  | x |  |  |  |  | | x |  |  |  |  | PANSS-Positive: | + | N/A | N/A | N/A |  |
| 47 | Priebe et al. (2015) | GBR |  | 179 | x | x |  |  |  |  | DIALOG+ | 6 (6) | AC |  |  | x |  |  |  |  | | x |  |  |  |  | PANSS-Positive: | - | - | N/A | N/A |  |
| 48 | Rathod et al. (2010) | GBR |  | 15 | x | x |  |  |  |  | CA-CBTp | - | - |  |  |  |  | x |  |  | |  |  |  |  |  | CBT or therapists help to understand the voices in a different and less stressful way. Voices should not be understood in a judgmental way. | | | | |  |
| 49 | Rathod et al. (2013) | GBR |  | 33 | x | x |  |  |  |  | CA-CBT-p | 16 (5) | TAU |  |  | x |  |  |  |  | |  |  |  |  | i | CPRS-AH Subscale | - | - | N/A | N/A |  |
| 50 ^c^ | Rosenbaum et al. (2012) | DNK |  | 269 | x | x |  |  |  |  | SPP-p | N/A (24) | TAU |  |  | x |  |  |  |  | | x |  |  |  |  | PANSS-Positive: | - | N/A | N/A | N/A |  |
| 51 | Schnackenberg et al. (2018) | DEU |  | 25^d^ |  |  | x |  | x |  | EFC | 30 (10) | AC |  |  |  |  | x |  |  | |  |  |  |  |  | EFC was considered helpful in understanding and working on trauma-related distress. | | | | |  |
| 52 ^c^ | Schnackenberg et al. (2016) | DEU |  | 12 |  |  | x |  |  |  | EFC | 30 (10) | AC |  |  | x |  |  |  | x | |  |  |  | x |  | BPRS-E Psychosis:  PSYRATS-AH: | -  - | +  - | 0.9  0.4 | 1.6  1.0 |  |
| 53 ^c^ | Schnackenberg et al. (2018b) | DEU |  | 25^d^ |  |  | x |  |  |  | EFC | 30 (10) | AC |  |  |  |  | x |  | |  |  |  |  |  |  | EFC was endorsed by professionals and voice hearers as a positive, not too stressful, with the potential to improve voice hearers’ lives overall. It was considered easy to implement and very helpful for most, especially to understand and working with the voices in a voice hearer´s life context. | | | | |  |
| 54 | Schnackenberg et al. (2014) | DEU | 0 | 0 |  |  | x |  | x |  | EFC | N/A | - | x |  |  |  |  | x |  | |  |  |  |  |  | There were no published intervention studies involving all elements of EFC/MsV. Two qualitative studies without a clear study design described MsV/EFC to help to understand voices within a person´s life context and promote personal recovery. | | | | |  |
| 55 | Singer et al. (2014) | CAN |  | 10 | x |  |  |  |  | Depr. | CBTp | 19 |  |  |  | x |  |  |  |  | | x |  |  |  |  | PANSS-Positive: | - | N/A | N/A | N/A |  |
| 56 ^c^ | Sivec et al. (2017) | USA |  | 6 | x | x | x | x |  |  | CBTp | 10 | TAU |  |  |  | CC |  |  | x | |  |  |  |  |  | Descriptive results: Very little differences related to PSYRATS-AH between the two groups. One individual in each group did not endorse hallucinations at post-test. | | | | |  |
| 57 | Sönmez et al. (2020) | NOR |  | 63 | x | x |  |  |  | Depr. | CBTp | 26 (6) | TAU |  |  | x |  |  |  |  | | x |  |  |  |  | PANSS-Positive: | - | | 0.30 | |  |
| 58 | Steel et al. (2019) | GBR |  | 15 |  |  | x |  |  |  | Making Sense of Voices (MsV) | 20 (9) | WL |  |  |  | CS |  |  | x | |  |  | x |  | j | PSYRATS-AH:  BAVQ-R subscales:  - malevolence  - benevolence  - omnipotence  DAIMON subscales:  - Person a. Voices  - Voices a. Person | -  -  -  -  -  - | -  -  -  +  -  - | 0.76  0.46  0.25  0.31  0.06  -0.04 | 1.57  0.46  0.08  0.78  -0.22  0.36 |  |
| 59 | Steel et al. (2020) | GBR |  | 12 |  |  | x |  |  |  | Making Sense of Voices | 20 (9) | WL |  |  |  |  | x |  |  | |  |  |  |  |  | Connection between positive outcomes and better understanding of the voice hearing experience and more control over the voices. Conversation with the voices was mostly considered possible but not experienced positively in some cases. | | | | |  |
| 60 | Stefaniak et al. (2019) | POL |  | 23 | x | x | x |  |  |  | AVATAR therapy | 8 (2) | TAU |  |  | x |  |  |  | x | |  |  |  |  | k | PSYRATS-AH  VPDS: | +  + | +  - | N/A  N/A | N/A  N/A |  |
| 61 | Tarrier et al. (2001) | GBR |  | 72 |  | x | x | x |  |  | CBTp | 20 (2.5) | AC & TAU |  |  | x |  |  |  |  | |  |  |  | x | l | PSE-Hallucinations: | + | N/A | N/A | N/A |  |
| 62 | Tarrier et al. (2014) | GBR |  | 49 |  | x |  |  |  | Suicidal | CBTp (CBSPp) | 24 (3) | TAU |  |  | x |  |  |  | x | | x |  |  |  |  | PANSS-Positive:  PSYRATS-AH: | +  - | +  - | N/A  N/A | 0.32  0.19 |  |
| 63 | Taylor et al. (2019) | GBR |  | 7 | x | x |  |  |  |  | CAT | N/A | - |  |  |  | CS | x |  |  | | x |  |  |  |  | Mixed-Methods:  Qualitative results: Very positive feedback from participants regarding understanding and dealing with voices.  Quantitative results: no measure for positive symptoms or voice hearing | | | | |  |
| 64 | Taylor et al. (2020) | GBR |  | 5 | x | x |  | x |  |  | iMAPS-Therapy | 6 |  |  |  |  | CS |  |  | x | |  |  |  |  |  | PANSS-Positive: PSYRATS-AH: | N/A  N/A | N/A  N/A | 1.45  1.36 | N/A  N/A |  |
| 65 | Temple & Ho (2005) | USA |  | 19 | x | x |  |  |  |  | CBTp | 20 | TAU |  |  |  | CT |  |  |  | |  | x |  |  |  | SAPS - Hallucinations: | - | N/A | 0.43 | N/A |  |
| 66 | Todorovic et al. (2020) | AUS | 4 | 571 | x | x |  |  |  |  | CBTp | 21-36 | AC & TAU | x | x |  |  |  |  | x | | x | x |  | x |  | Positive Symptoms:  PSYRATS-AH subscales:  - physical  - emotional  - cognitive | +  +  +  - | | 0.33  0.29  0.25  0.26 | |  |
| 67 | Turkington et al. (2006) | GBR |  | 422 | x | x |  |  |  |  | CBTp | N/A | TAU |  |  | x |  |  |  | x | |  |  |  |  |  | PSYRATS-AH: | N/A | - | N/A | N/A |  |
| 68 | Turkington et al. (2014) | GBR |  | 38 | x | x | x | x |  |  | CBTp  (HYCBt-p) | 12 | - |  |  |  | OS |  |  | x | |  |  |  |  | i | PSYRATS-AH: | - | N/A | 0.58 | N/A |  |
| 69 | Turner et al. (2014) | NLD | 48 | 3’295 | x | x |  |  |  |  | Psychological Interventions for psychosis | (1-24) | AC | x | x |  |  |  |  | x | | x | x | x | x |  | CBTp vs SC:  Positive Symptoms: | + | | 0.23 | |  |
|  |  |  |  |  |  |  |  |  |  |  |  |  |  |  |  |  |  |  |  |  |  |  |  |  |  |  | CBTp vs any other intervention: slight superiority in reducing positive symptoms. Individual setting slightly superior to group. | | | | |  |
| 70 | Utzenoff et al. (2008) | USA |  | 24 | x | x |  |  |  |  | ACE | 14 (6) | AC |  |  | x |  |  |  |  | | x |  |  |  |  | PANSS-Positive: | + | N/A | N/A | N/A |  |
| 71 | van den Berg et al. (2018) | NDL |  | 155 | x | x |  |  | x | PTSD | EMDR or PE | 10 (8) | WL |  |  | x |  |  |  | x | |  |  |  |  |  | PSYRATS-AH: | - | - | N/A | N/A |  |
| 72 | van der Gaag et al. (2011) | NDL |  | 216 | x |  |  |  |  |  | CBTp | 26 (7) | TAU |  |  | x |  |  |  | x | | x |  |  |  |  | PSYRATS-Total: | + | N/A | N/A | N/A |  |
| 73 ^c^ | Varese et al. (2020) | GBR |  | 19 | x | x | x |  | x | Diss. | CBTp | 24 (6) | - |  |  |  | CS |  |  | x | |  |  |  |  |  | PSYRATS-AH:  (within-group analysis) | + | + | 0.65 | 1.08 |  |
| 74 | Velligan et al. (2015) | GBR |  | 166 | x | x | x | x |  |  | CBTp or CAT | 38 (9) | AC or TAU |  |  | x |  |  |  | x | |  |  |  | x |  | CAT vs not CAT PSYRATS-AH: | + | | 0.36 | |  |
| 75 | Waller et al. (2018) | GBR |  | 75 | x |  |  |  |  | Anx. & Depr. | CBTp (GOALS) | 8 (2) | TAU |  |  | x |  |  |  | x | | x |  |  |  |  | PANSS-Positive:  PSYRATS-AH | -  - | -  - | 0.07  0.04 | 0.13  0.04 |  |
| 76 | Wykes et al. (2008) | GBR | 27 | 1’565 | x | x |  |  |  |  | CBTp | N/A | N/A | x | x |  |  |  |  |  | | x |  |  | x |  | Positive Symptoms: | + | | 0.37 | | |
| 77 | Yildiz et al. (2019) | TUR | 11 | 473 | x | x |  |  |  |  | ACT | 3-10 (x-12) | AC or TAU | x |  |  |  |  |  | x | | x |  | x | x |  | Positive Symptoms:  Hearing Voices: | +  + | +  - | N/A  N/A | N/A  N/A | |

**^a^** **Abbreviations:** **AC** = Active Control; **ACE** = Adherence-Coping-Education; **ACT** = Acceptance and Commitment Therapy; **Anx.**: Anxiety; **BPRS** = Brief Psychiatric Rating Scale; **BPRS-E** = Brief Psychiatric Rating Scale -Expanded Version; **CA-CBTp** = culturally adapted Cognitive Behavioural Therapy for Psychosis; **CAT** = Cognitive Analytic Therapy; CBFI: Cognitive Behavioural Family Intervention; **CBSPp** = cognitive behavioural prevention of suicide in psychosis; **CBT** = Cognitive Behavioural Therapy; **CBTp** = Cognitive Behavioural Therapy for Psychosis; **CC** = (retrospective) Case Comparison; **CFT** = Coping Focussed Therapy; **CNI** = Cognitive Nursing Intervention; **CR** = Cognitive Remediation; **CS** = Case Series; **CT** = Controlled Study; **Depr.** = Depression; **Diss.**: Dissociation; **EMDR** = Eye Movement Desensitization and Reprocessing; **FS** = Feasibility Study; **GOALS** = Getting On top of Anxiety and Low Mood and So reaching your goals; **GRIP** = Graduated Recovery Intervention; HPSVQ = Hamilton Program for Schizophrenia Voices Questionnaire (HPSVQ); **HYCBt-p** = High-Yield Cognitive Behavioural Techniques for Psychosis; **iMAPS** = Imagery focused psychological therapy for persecutory delusions in Psychosis; **MA** = Meta-Analysis; **MBCI** = Mindfulness-based crisis interventions; **MBCT**: Mindfulness Based Cognitive Intervention; **MCT** = Meta-cognitive Training; **MI** = Motivational Interviewing; **MS** = Meta synthesis **OS** = Observational Study; **PANSS** =Positive and Negative Syndrome Scale; **PE** = Prolonged Exposure; **PSYRATS** = Psychotic Symptom Rating Scales; **PTSD**: Post Traumatic Stress Disorder; **RCT** = Randomized Controlled Trial; **SAPS** = Scale for the Assessment of Positive Symptoms; **SAT** = Social Activation Therapy; **SC** = Supportive Counselling; **SPP** = Supportive Psychodynamic Psychotherapy; **SPPp**: Supportive Psychodynamic Psychotherapy for Psychosis; **SR** = Systematic Review; **TAU** =Treatment As Usual; **TE** = Training Evaluation; **TF-CBTp** = trauma-focused Cognitive Behavioural Therapy for Psychosis; THC = Cannabis; **VAY** = Voice and You – Questionnaire; **VRE** = Virtual Reality Exposure; **WL** = Waitlist; **WS** = Within-Subject Design (2 [Time: pre vs. post] × 3 (Condition: VT, AT and CC) repeated measure.

**^b^ Outcome measures – others (few named)**: **a** = Visual Analog Scale (VAS) - Coping with and understanding voices; **b** = Behaviour and Symptom Identification Scale (BASIS-24) - Hallucination Items; **c** = Voice and You – Questionnaire (VAY); **d** = Self-rating of psychotic symptoms (Voices): frequency, distress, believability; **e** = Hamilton Program for Schizophrenia Voices Questionnaire (HPSVQ); **f** = Community Assessment of Psychic Experience (CAPE) questionnaire; **g** = Voice Compliance Scale (VCS); **h** = Subjective Units of Disturbance (SUD). **i** = CPRS-Schizophrenia change Subscale; **j** = DIAMON-Scale; **k** = Voice Power Differential Scale (VPDS); **l** = Present State Examination (PSE), hallucination symptoms categories

**^c^** Studies in which nurses or other health professions were named as interventionist

**^d^** Including 11 professionals as participants which were part of the qualitative studies

# References of all included studies in the review ( n=77)

1. Aali G, Kariotis T, Shokraneh F. Avatar Therapy for People with Schizophrenia or Related Disorders. *Cochrane Database Syst Rev* (2020) 5(5):Cd011898. Epub 2020/05/16. doi: 10.1002/14651858.CD011898.pub2.

2. Adams R, Ohlsen S, Wood E. Eye Movement Desensitization and Reprocessing (Emdr) for the Treatment of Psychosis: A Systematic Review. *Eur J Psychotraumatol* (2020) 11(1):1711349. Epub 2020/04/15. doi: 10.1080/20008198.2019.1711349.

3. Bacon T, Farhall J, Fossey E. The Active Therapeutic Processes of Acceptance and Commitment Therapy for Persistent Symptoms of Psychosis: Clients' Perspectives. *Behav Cogn Psychother* (2014) 42(4):402-20. doi: http://dx.doi.org/10.1017/S1352465813000209.

4. Balzan RP, Delfabbro PH, Galletly CA, Woodward TS. Metacognitive Training for Patients with Schizophrenia: Preliminary Evidence for a Targeted, Single-Module Programme. *Aust N Z J Psychiatry* (2014) 48(12):1126-36. doi: 10.1177/0004867413508451.

5. Balzan RP, Mattiske JK, Delfabbro P, Liu D, Galletly C. Individualized Metacognitive Training (Mct+) Reduces Delusional Symptoms in Psychosis: A Randomized Clinical Trial. *Schizophr Bull* (2019) 45(1):27‐36. doi: 10.1093/schbul/sby152.

6. Barrowclough C, Haddock G, Wykes T, Beardmore R, Conrod P, Craig T, et al. Integrated Motivational Interviewing and Cognitive Behavioural Therapy for People with Psychosis and Comorbid Substance Misuse: Randomised Controlled Trial. *BMJ* (Clinical research ed) (2010) 341:c6325. doi: 10.1136/bmj.c6325.

7. Barrowclough C, Marshall M, Gregg L, Fitzsimmons M, Tomenson B, Warburton J, et al. A Phase-Specific Psychological Therapy for People with Problematic Cannabis Use Following a First Episode of Psychosis: A Randomized Controlled Trial. *Psychol Med* (2014) 44(13):2749-61. doi: http://dx.doi.org/10.1017/S0033291714000208.

8. Beames L, Strodl E, Dark F, Wilson J, Sheridan J, Kerswell N. A Feasibility Study of the Translation of Cognitive Behaviour Therapy for Psychosis into an Australian Adult Mental Health Clinical Setting. *Behav Change* (2020) 37(1):22-32. doi: http://dx.doi.org/10.1017/bec.2020.1.

9. Bell IH, Rossell SL, Farhall J, Hayward M, Lim MH, Fielding-Smith SF, et al. Pilot Randomised Controlled Trial of a Brief Coping-Focused Intervention for Hearing Voices Blended with Smartphone-Based Ecological Momentary Assessment and Intervention (Savvy): Feasibility, Acceptability and Preliminary Clinical Outcomes. *Schizophr Res* (2020) 216:479-87. doi: http://dx.doi.org/10.1016/j.schres.2019.10.026.

10. Berry C, Hayward M. What Can Qualitative Research Tell Us About Service User Perspectives of Cbt for Psychosis? A Synthesis of Current Evidence. *Behav Cogn Psychother* (2011) 39(4):487-94. Epub 2011/04/05. doi: 10.1017/s1352465811000154.

11. Birchwood M, Mohan L, Meaden A, Tarrier N, Lewis S, Wykes T, et al. The Command Trial of Cognitive Therapy for Harmful Compliance with Command Hallucinations (Ctch): A Qualitative Study of Acceptability and Tolerability in the Uk. *BMJ open* (2018) 8(6):e021657. Epub 2018/06/18. doi: 10.1136/bmjopen-2018-021657.

12. Brand RM, McEnery C, Rossell S, Bendall S, Thomas N. Do Trauma-Focussed Psychological Interventions Have an Effect on Psychotic Symptoms? A Systematic Review and Meta-Analysis. *Schizophr Res* (2018) 195:13-22. Epub 2017/08/29. doi: 10.1016/j.schres.2017.08.037.

13. Brown E, Shrestha M, Gray R. The Safety and Efficacy of Acceptance and Commitment Therapy against Psychotic Symptomatology: A Systematic Review and Meta-Analysis. *Braz J Psychiatry* (2020). Epub 2020/08/07. doi: 10.1590/1516-4446-2020-0948.

14. Buck B, Norr A, Katz A, Gahm GA, Reger GM. Reductions in Reported Persecutory Ideation and Psychotic-Like Experiences During Exposure Therapy for Posttraumatic Stress Disorder. *Psychiatr Res* (2019) 272:190-5. doi: http://dx.doi.org/10.1016/j.psychres.2018.12.022.

15. Burns AM, Erickson DH, Brenner CA. Cognitive-Behavioral Therapy for Medication-Resistant Psychosis: A Meta-Analytic Review. *Psychiatr Serv* (2014) 65(7):874-80. Epub 2014/04/02. doi: 10.1176/appi.ps.201300213.

16. Drake RJ, Day CJ, Picucci R, Warburton J, Larkin W, Husain N, et al. A Naturalistic, Randomized, Controlled Trial Combining Cognitive Remediation with Cognitive-Behavioural Therapy after First-Episode Non-Affective Psychosis. *Psychol Med* (2014) 44(9):1889-99. doi: http://dx.doi.org/10.1017/S0033291713002559.

17. England M. Efficacy of Cognitive Nursing Intervention for Voice Hearing. *Perspect Psychiatr Care* (2007) 43(2):69-76. doi: 10.1111/j.1744-6163.2007.00114.x.

18. Farhall J, Freeman NC, Shawyer F, Trauer T. An Effectiveness Trial of Cognitive Behaviour Therapy in a Representative Sample of Outpatients with Psychosis. *Br J Clin Psychol* (2009) 48(Pt 1):47-62. Epub 2008/10/15. doi: 10.1111/j.2044-8260.2009.tb00456.x.

19. Fichtenbauer I, Priebe S, Schrank B. Die Deutsche Version Von Dialog+ Bei Patientinnen Mit Psychose - Eine Pilotstudie., the German Version of Dialog+ for Patients with Psychosis - a Pilot Study. *Psychiatr Prax* (2019) 46(7):376-80. doi: http://dx.doi.org/10.1055/a-0961-3328.

20. Garety PA, Fowler DG, Freeman D, Bebbington P, Dunn G, Kuipers E. Cognitive--Behavioural Therapy and Family Intervention for Relapse Prevention and Symptom Reduction in Psychosis: Randomised Controlled Trial. *Br J Psychiatry* (2008) 192(6):412-23. Epub 2008/06/03. doi: 10.1192/bjp.bp.107.043570.

21. Gould RA, Mueser KT, Bolton E, Mays V, Goff D. Cognitive Therapy for Psychosis in Schizophrenia: An Effect Size Analysis. *Schizophr Res* (2001) 48(2-3):335-42. Epub 2001/04/11. doi: 10.1016/s0920-9964(00)00145-6.

22. Haddock G, Barrowclough C, Shaw JJ, Dunn G, Novaco RW, Tarrier N. Cognitive-Behavioural Therapy V. Social Activity Therapy for People with Psychosis and a History of Violence: Randomised Controlled Trial. *Br J Psychiatry* (2009) 194(2):152-7. doi: http://dx.doi.org/10.1192/bjp.bp.107.039859.

23. Harder S, Koester A, Valbak K, Rosenbaum B. Five-Year Follow-up of Supportive Psychodynamic Psychotherapy in First-Episode Psychosis: Long-Term Outcome in Social Functioning. *Psychiatry* (2014) 77(2):155-68. Epub 2014/05/29. doi: 10.1521/psyc.2014.77.2.155.

24. Hayward M, Jones AM, Bogen-Johnston L, Thomas N, Strauss C. Relating Therapy for Distressing Auditory Hallucinations: A Pilot Randomized Controlled Trial. *Schizophr Res* (2017) 183:137-42. Epub 2016/12/06. doi: 10.1016/j.schres.2016.11.019.

25. Hayward M, Overton J, Dorey T, Denney J. Relating Therapy for People Who Hear Voices: A Case Series. *Clin Psychol Psychother* (2009) 16(3):216-27. Epub 2009/05/21. doi: 10.1002/cpp.615.

26. Husain MO, Chaudhry IB, Mehmood N, Rehman RU, Kazmi A, Hamirani M, et al. Pilot Randomised Controlled Trial of Culturally Adapted Cognitive Behavior Therapy for Psychosis (Cacbtp) in Pakistan. *BMC Health Serv Res* (2017) 17(1):808. doi: http://dx.doi.org/10.1186/s12913-017-2740-z.

27. Jacobsen P, Peters E, Robinson EJ, Chadwick P. Mindfulness-Based Crisis Interventions (Mbci) for Psychosis within Acute Inpatient Psychiatric Settings; a Feasibility Randomised Controlled Trial. *BMC Psychiatry* (2020) 20(1):193. Epub 2020/05/01. doi: 10.1186/s12888-020-02608-x.

28. Jolley S, Onwumere J, Bissoli S, Bhayani P, Singh G, Kuipers E, et al. A Pilot Evaluation of Therapist Training in Cognitive Therapy for Psychosis: Therapy Quality and Clinical Outcomes. *Behav Cogn Psychother* (2015) 43(4):478-89. doi: http://dx.doi.org/10.1017/S1352465813001100.

29. Keen N, Hunter ECM, Peters E. Integrated Trauma-Focused Cognitive-Behavioural Therapy for Post-Traumatic Stress and Psychotic Symptoms: A Case-Series Study Using Imaginal Reprocessing Strategies. *Front Psychiatr* (2017) 8(JUN):92. doi: http://dx.doi.org/10.3389/fpsyt.2017.00092.

30. Klingberg S, Herrlich J, Wiedemann G, Wölwer W, Meisner C, Engel C, et al. Adverse Effects of Cognitive Behavioral Therapy and Cognitive Remediation in Schizophrenia: Results of the Treatment of Negative Symptoms Study. *J Nerv Ment Dis* (2012) 200(7):569-76. doi: 10.1097/nmd.0b013e31825bfa1d.

31. Kråkvik B, Gråwe RW, Hagen R, Stiles TC. Cognitive Behaviour Therapy for Psychotic Symptoms: A Randomized Controlled Effectiveness Trial. *Behav Cogn Psychother* (2013) 41(5):511‐24. doi: 10.1017/S1352465813000258.

32. Lamster F, Kiener J, Wagner K, Rief W, Goerge SC, Iwaniuk S, et al. Are Delusions Indirectly Changeable? A Mood-Enhancing Concept of Cognitive Behavioural Therapy for Standard Inpatient Care of Patients with Schizophrenic Disorders [Ist Wahn Indirekt Veraenderbar? Ein Stimmungsverbesserndes Konzept Der Kognitive Verhaltenstherapie Fuer Die Stationaere Standardversorgung Von Patienten Mit Schizophrenen Stoerungen]. *Verhaltenstherapie* (2018) 28(3):138-46. doi: http://dx.doi.org/10.1159/000486966.

33. Lewis S, Tarrier N, Haddock G, Bentall R, Kinderman P, Kingdon D, et al. Randomised Controlled Trial of Cognitive-Behavioural Therapy in Early Schizophrenia: Acute-Phase Outcomes. *Br J Psychiatry Suppl* (2002) 43:s91‐7. doi: 10.1192/bjp.181.43.s91.

34. Lincoln TM, Jung E, Wiesjahn M, Schlier B. What Is the Minimal Dose of Cognitive Behavior Therapy for Psychosis? An Approximation Using Repeated Assessments over 45 Sessions. [Was Ist Die Minimale Dosis Der Kognitiven Verhaltenstherapie Fuer Psychose? Eine Annaeherung Mittels Wiederholter Messungen Ueber 45 Sitzungen]. *Eur Psychiatry* (2016) 38:31-9. doi: http://dx.doi.org/10.1016/j.eurpsy.2016.05.004.

35. Lincoln TM, Peters E. A Systematic Review and Discussion of Symptom Specific Cognitive Behavioural Approaches to Delusions and Hallucinations. *Schizophr Res* (2019) 203:66-79. doi: https://doi.org/10.1016/j.schres.2017.12.014.

36. Lincoln TM, Ziegler M, Mehl S, Kesting M-L, Luellmann E, Westermann S, et al. Moving from Efficacy to Effectiveness in Cognitive Behavioral Therapy for Psychosis: A Randomized Clinical Practice Trial. [Von Der Wirksamkeit Der Kognitiven Verhaltenstherapie Bei Psychose Unter Kontrollierten Bedingungen Hin Zur Wirksamkeit in Der Praktischen Anwendung: Eine Randomisierte Klinische Praxisstudie]. *J Consult Clin Psychol* (2012) 80(4):674-86. doi: http://dx.doi.org/10.1037/a0028665.

37. Louise S, Rossell SL, Thomas N. The Acceptability, Feasibility and Potential Outcomes of an Individual Mindfulness-Based Intervention for Hearing Voices. *Behav Cogn Psychother* (2019) 47(2):200-16. Epub 2018/07/10. doi: 10.1017/s1352465818000425.

38. Ma CF, Chan SKW, Chien WT, Bressington D, Mui EYW, Lee EHM, et al. Cognitive Behavioural Family Intervention for People Diagnosed with Severe Mental Illness and Their Families: A Systematic Review and Meta-Analysis of Randomized Controlled Trials. *J Psychiatr Ment Health Nurs* (2020) 27(2):128-39. Epub 2019/09/25. doi: 10.1111/jpm.12567.

39. Matthijssen SJMA, Heitland I, Verhoeven LCM, van den Hout MA. Reducing the Emotionality of Auditory Hallucination Memories in Patients Suffering from Auditory Hallucinations., Reduzierung Der Emotionalitaet Der Auditiven Halluzinationserinnerungen Bei Patienten, Die an Auditiven Halluzinationen Leiden (Deepl) (Psyndexshort). *Front Psychiatr* (2019) 10:637. doi: http://dx.doi.org/10.3389/fpsyt.2019.00637.

40. Morrison AP, Hutton P, Wardle M, Spencer H, Barratt S, Brabban A, et al. Cognitive Therapy for People with a Schizophrenia Spectrum Diagnosis Not Taking Antipsychotic Medication: An Exploratory Trial. *Psychol Med* (2012) 42(5):1049-56. doi: 10.1017/S0033291711001899.

41. Morrison AP, Pyle M, Chapman N, French P, Parker SK, Wells A. Metacognitive Therapy in People with a Schizophrenia Spectrum Diagnosis and Medication Resistant Symptoms: A Feasibility Study. *J Behav Ther Exp Psychiatry* (2014) 45(2):280-4. doi: 10.1016/j.jbtep.2013.11.003.

42. Morrison AP, Renton JC, Williams S, Dunn H, Knight A, Kreutz M, et al. Delivering Cognitive Therapy to People with Psychosis in a Community Mental Health Setting: An Effectiveness Study. *Acta Psychiatr Scand* (2004) 110(1):36-44. Epub 2004/06/08. doi: 10.1111/j.1600-0447.2004.00299.x.

43. Morrison AP, Turkington D, Pyle M, Spencer H, Brabban A, Dunn G, et al. Cognitive Therapy for People with Schizophrenia Spectrum Disorders Not Taking Antipsychotic Drugs: A Single-Blind Randomised Controlled Trial. *Lancet, North American Edition* (9926):1395-403. doi: 10.1016/S0140-6736(13)62246-1.

44. Penn DL, Uzenoff SR, Perkins D, Mueser KT, Hamer R, Waldheter E, et al. A Pilot Investigation of the Graduated Recovery Intervention Program (Grip) for First Episode Psychosis. *Schizophr Res* (2011) 125(2):247-56. doi: https://doi.org/10.1016/j.schres.2010.08.006.

45. Peters E, Crombie T, Agbedjro D, Johns LC, Stahl D, Greenwood K. The Long-Term Effectiveness of Cognitive Behavior Therapy for Psychosis within a Routine Psychological Therapies Service. *Front Psychol* (2015) 6:1658. doi: 10.3389/fpsyg.2015.01658.

46. Premkumar P, Peters ER, Fannon D, Anilkumar AP, Kuipers E, Kumari V. Coping Styles Predict Responsiveness to Cognitive Behaviour Therapy in Psychosis. *Psychiatr Res* (2011) 187(3):354-62. doi: http://dx.doi.org/10.1016/j.psychres.2010.12.029.

47. Priebe S, Kelley L, Omer S, Golden E, Walsh S, Khanom H, et al. The Effectiveness of a Patient-Centred Assessment with a Solution-Focused Approach (Dialog+) for Patients with Psychosis: A Pragmatic Cluster-Randomised Controlled Trial in Community Care. *Psychother Psychosom* (2015) 84(5):304‐13. doi: 10.1159/000430991.

48. Rathod S, Kingdon D, Phiri P, Gobbi M. Developing Culturally Sensitive Cognitive Behaviour Therapy for Psychosis for Ethnic Minority Patients by Exploration and Incorporation of Service Users' and Health Professionals' Views and Opinions. *Behav Cogn Psychother* (2010) 38(5):511-33.

49. Rathod S, Phiri P, Harris S, Underwood C, Thagadur M, Padmanabi U, et al. Cognitive Behaviour Therapy for Psychosis Can Be Adapted for Minority Ethnic Groups: A Randomised Controlled Trial. *Schizophr Res* (2013) 143(2/3):319-26. doi: 10.1016/j.schres.2012.11.007.

50. Rosenbaum B, Harder S, Knudsen P, Køster A, Lindhardt A, Lajer M, et al. Supportive Psychodynamic Psychotherapy Versus Treatment as Usual for First-Episode Psychosis: Two-Year Outcome. *Psychiatry* (2012) 75(4):331-41. Epub 2012/12/19. doi: 10.1521/psyc.2012.75.4.331.

51. Schnackenberg J, Fleming M, Martin CR. A Randomised Controlled Pilot Study of Experience Focused Counselling with Voice Hearers. *Psychosis* (2016):1-13. doi: 10.1080/17522439.2016.1185452.

52. Schnackenberg J, Fleming M, Walker H, Martin CR. Experience Focussed Counselling with Voice Hearers: Towards a Trans-Diagnostic Key to Understanding Past and Current Distress—a Thematic Enquiry. *Community Ment Health J* (2018) 54(7):1071-81. doi: 10.1007/s10597-018-0280-6.

53. Schnackenberg J, Martin CR. The Need for Experience Focused Counselling (Efc) with Voice Hearers in Training and Practice: A Review of the Literature. *J Psychiatr Ment Health Nurs* (2014) 21(5):391-402. doi: 10.1111/jpm.12084.

54. Schnackenberg JK, Fleming M, Martin CR. Experience Focussed Counselling with Voice Hearers as a Trauma-Sensitive Approach. Results of a Qualitative Thematic Enquiry. *Community Ment Health J* (2018) 54(7):997-1007. doi: 10.1007/s10597-018-0294-0.

55. Singer AR, Addington DE, Dobson KS, Wright C. A Pilot Study of Cognitive Behavior Therapy for Depression in Early Psychosis. *Cogn Behav Pract* (2014) 21(3):323-34. doi: http://dx.doi.org/10.1016/j.cbpra.2013.08.004.

56. Sivec HJ, Montesano VL, Skubby D, Knepp KA, Munetz MR. Cognitive Behavioral Therapy for Psychosis (Cbt-P) Delivered in a Community Mental Health Setting: A Case Comparison of Clients Receiving Cbt Informed Strategies by Case Managers Prior to Therapy. *Community Ment Health J* (2017) 53(2):134-42. doi: http://dx.doi.org/10.1007/s10597-015-9930-0.

57. Sonmez N, Romm KL, Ostefjells T, Grande M, Jensen LH, Hummelen B, et al. Cognitive Behavior Therapy in Early Psychosis with a Focus on Depression and Low Self-Esteem: A Randomized Controlled Trial. *Compr Psychiatry* (2020) 97:152157. doi: http://dx.doi.org/10.1016/j.comppsych.2019.152157.

58. Steel C, Schnackenberg J, Perry H, Longden E, Greenfield E, Corstens D. Making Sense of Voices: A Case Series. *Psychosis* (2019) 11(1):3-15. doi: 10.1080/17522439.2018.1559874.

59. Steel C, Schnackenberg J, Travers Z, Longden E, Greenfield E, Meredith L, et al. Voice Hearers’ Experiences of the Making Sense of Voices Approach in an Nhs Setting. *Psychosis* (2020) 12(2):106-14. doi: 10.1080/17522439.2019.1707859.

60. Stefaniak I, Sorokosz K, Janicki A, Wciorka J. Therapy Based on Avatar-Therapist Synergy for Patients with Chronic Auditory Hallucinations: A Pilot Study. *Schizophr Res* (2019) 211:115‐7. doi: 10.1016/j.schres.2019.05.036.

61. Tarrier N, Kelly J, Maqsood S, Snelson N, Maxwell J, Law H, et al. The Cognitive Behavioural Prevention of Suicide in Psychosis: A Clinical Trial. *Schizophr Res* (2014) 156(2-3):204-10. Epub 2014/05/24. doi: 10.1016/j.schres.2014.04.029.

62. Tarrier N, Kinney C, McCarthy E, Wittkowski A, Yusupoff L, Gledhill A, et al. Are Some Types of Psychotic Symptoms More Responsive to Cognitive-Behaviour Therapy? *Behav Cogn Psychother* (2001) 29(1):45-55. doi: http://dx.doi.org/10.1017/S1352465801001060.

63. Taylor CDJ, Bee PE, Kelly J, Emsley R, Haddock G. Imagery Focused Psychological Therapy for Persecutory Delusions in Psychosis (Imaps): A Multiple Baseline Experimental Case Series. *Behav Cogn Psychother* (2020) 48(5):530-45. doi: http://dx.doi.org/10.1017/S1352465820000168.

64. Taylor PJ, Perry A, Hutton P, Tan R, Fisher N, Focone C, et al. Cognitive Analytic Therapy for Psychosis: A Case Series. *Psychol Psychother* (2019) 92(3):359-78. doi: http://dx.doi.org/10.1111/papt.12183.

65. Temple S, Ho BC. Cognitive Therapy for Persistent Psychosis in Schizophrenia: A Case-Controlled Clinical Trial. *Schizophr Res* (2005) 74(2‐3):195‐9. doi: 10.1016/j.schres.2004.05.013.

66. Todorovic A, Lal S, Dark F, De Monte V, Kisely S, Siskind D. Cbtp for People with Treatment Refractory Schizophrenia on Clozapine: A Systematic Review and Meta-Analysis. *J Ment Health* (2020):1-8. Epub 2020/10/21. doi: 10.1080/09638237.2020.1836558.

67. Turkington D, Kingdon D, Rathod S, Hammond K, Pelton J, Mehta R. Outcomes of an Effectiveness Trial of Cognitive-Behavioural Intervention by Mental Health Nurses in Schizophrenia. *Br J Psychiatry* (2006) 189:36-40. Epub 2006/07/04. doi: 10.1192/bjp.bp.105.010884.

68. Turkington D, Munetz M, Pelton J, Montesano V, Sivec H, Nausheen B, et al. High-Yield Cognitive Behavioral Techniques for Psychosis Delivered by Case Managers to Their Clients with Persistent Psychotic Symptoms: An Exploratory Trial. *J Nerv Ment Dis* (2014) 202(1):30-4. Epub 2014/01/01. doi: 10.1097/nmd.0000000000000070.

69. Turner DT, van der Gaag M, Karyotaki E, Cuijpers P. Psychological Interventions for Psychosis: A Meta-Analysis of Comparative Outcome Studies. *Am J Psychiatry* (2014) 171(5):523-38. Epub 2014/02/15. doi: 10.1176/appi.ajp.2013.13081159.

70. Uzenoff SR, Perkins DO, Hamer RM, Wiesen CA, Penn DL. A Preliminary Trial of Adherence-Coping-Education (Ace) Therapy for Early Psychosis. *J Nerv Ment Dis* (2008) 196(7):572-5. doi: http://dx.doi.org/10.1097/NMD.0b013e31817d01a5.

71. van den Berg D, de Bont P, van der Vleugel BM, de Roos C, de Jongh A, van Minnen A, et al. Long-Term Outcomes of Trauma-Focused Treatment in Psychosis. *Br J Psychiatry* (2018) 212(3):180-2. Epub 2018/02/14. doi: 10.1192/bjp.2017.30.

72. van der Gaag M, Stant AD, Wolters KJ, Buskens E, Wiersma D. Cognitive-Behavioural Therapy for Persistent and Recurrent Psychosis in People with Schizophrenia-Spectrum Disorder: Cost-Effectiveness Analysis. *Br J Psychiatry* (2011) 198(1):59-65, sup 1. Epub 2011/01/05. doi: 10.1192/bjp.bp.109.071522.

73. Varese F, Douglas M, Dudley R, Bowe S, Christodoulides T, Common S, et al. Targeting Dissociation Using Cognitive Behavioural Therapy in Voice Hearers with Psychosis and a History of Interpersonal Trauma: A Case Series. *Psychol Psychother* (2020):e12304. doi: http://dx.doi.org/10.1111/papt.12304.

74. Velligan DI, Tai S, Roberts DL, Maples-Aguilar N, Brown M, Mintz J, et al. A Randomized Controlled Trial Comparing Cognitive Behavior Therapy, Cognitive Adaptation Training, Their Combination and Treatment as Usual in Chronic Schizophrenia. *Schizophr Bull* (2015) 41(3):597-603. Epub 2014/09/07. doi: 10.1093/schbul/sbu127.

75. Waller H, Landau S, Fornells-Ambrojo M, Jolley S, McCrone P, Halkoree R, et al. Improving Implementation of Evidence Based Practice for People with Psychosis through Training the Wider Workforce: Results of the Goals Feasibility Randomised Controlled Trial. *J Behav Ther Exp Psychiatry* (2018) 59:121‐8. doi: 10.1016/j.jbtep.2017.12.004.

76. Wykes T, Steel C, Everitt B, Tarrier N. Cognitive Behavior Therapy for Schizophrenia: Effect Sizes, Clinical Models, and Methodological Rigor. *Schizophr Bull* (2008) 34(3):523-37. Epub 2007/10/27. doi: 10.1093/schbul/sbm114.

77. Yıldız E. The Effects of Acceptance and Commitment Therapy in Psychosis Treatment: A Systematic Review of Randomized Controlled Trials. *Perspect Psychiatr Care* (2020) 56(1):149-67. Epub 2019/05/11. doi: 10.1111/ppc.12396.
